# Modelling the Human Blood–Brain Barrier in Huntington Disease

**DOI:** 10.3390/ijms23147813

**Published:** 2022-07-15

**Authors:** Domenico Vignone, Odalys Gonzalez Paz, Ivan Fini, Antonella Cellucci, Giulio Auciello, Maria Rosaria Battista, Isabelle Gloaguen, Silvia Fortuni, Cristina Cariulo, Vinod Khetarpal, Celia Dominguez, Ignacio Muñoz-Sanjuán, Annalise Di Marco

**Affiliations:** 1 IRBM SpA, Via Pontina km 30,600, 00071 Pomezia, Italy; d.vignone@irbm.com (D.V.); o.gonzalez@irbm.com (O.G.P.); i.fini@irbm.com (I.F.); a.cellucci@irbm.com (A.C.); g.auciello@irbm.com (G.A.); m.battista@irbm.com (M.R.B.); gloaguenisa@gmail.com (I.G.); s.fortuni@irbm.com (S.F.); c.cariulo@irbm.com (C.C.); 2 CHDI Management/CHDI Foundation, 6080 Center Drive, Los Angeles, CA 90045, USA; vinod.khetarpal@chdifoundation.org (V.K.); celia.dominguez@chdifoundation.org (C.D.); ignacio.munoz@chdifoundation.org (I.M.-S.)

**Keywords:** blood–brain barrier, transport, induced pluripotent stem cells, brain endothelial cells, in vitro models, Huntington’s disease

## Abstract

While blood–brain barrier (BBB) dysfunction has been described in neurological disorders, including Huntington’s disease (HD), it is not known if endothelial cells themselves are functionally compromised when promoting BBB dysfunction. Furthermore, the underlying mechanisms of BBB dysfunction remain elusive given the limitations with mouse models and post mortem tissue to identify primary deficits. We established models of BBB and undertook a transcriptome and functional analysis of human induced pluripotent stem cell (iPSC)-derived brain-like microvascular endothelial cells (iBMEC) from HD patients or unaffected controls. We demonstrated that HD-iBMECs have abnormalities in barrier properties, as well as in specific BBB functions such as receptor-mediated transcytosis.

## 1. Introduction

Huntington’s disease (HD) is an autosomal dominant neurodegenerative disorder caused by the CAG repeat in the exon 1 of the huntingtin gene, which encodes for huntingtin (HTT), a cytoplasmic protein ubiquitously expressed in all cells of the body. It is believed that mutant huntingtin (mHTT) protein causes dysfunction and death in basal ganglia neurons, which leads to a progressive disorder of movement and cognition. Although the disease has long been considered a disorder of the brain, abnormalities outside the central nervous system (CNS) are also commonly observed in HD [1,2]. Changes in cerebrovascular vessel density in patients with HD as well as in transgenic mouse models of the disease have been reported [3]. Moreover, the expression of mHTT within the neurovascular components and the morphological and functional changes in cerebral blood vessels have been observed in R6/mice and in mild-to-moderate stage HD patients, as well as in post mortem tissues [4,5,6]. The dysfunction of the BBB, such as the impairment of tight junctions (TJs) formation and function, has also been reported to be associated with other neurodegenerative diseases such as Alzheimer’s (AD) and Parkinson’s (PD), especially in the late stages of the disease [7].

Our current research comprises examining how BBB impairment contributes to disease progression in CNS diseases. Moreover, the BBB remains a major obstacle to pharmaceutical intervention in CNS research and the understanding of functional characteristics using in vitro platforms might help elucidate disease mechanisms and identify potential targets for delivery and therapeutic modulation of the BBB. As a promising in vitro approach, brain endothelial cells derived from *human*-induced pluripotent stem cells (iPSCs) have been demonstrated to possess major characteristics of the in vivo BBB. During recent years, there has been a rapid increase in iPSC-derived BBB models used as tools in the investigations of drug permeability [8,9,10,11] and disease modelling [7,12,13].

The ideal in vitro BBB model should display several criteria: the presence of the key transport mechanisms responsible for the selective entry of nutrients, long-term preservation of the structural and functional integrity during culturing, the reproduction of specific properties of the defined cell population within the model in physiological and pathophysiological conditions, and relative ease in conducting the transport studies in a drug discovery setting.

There are three critical characteristics of brain endothelium that establish this barrier: (1) TJs that restrict the diffusion of molecules, (2) a small number of endocytotic vesicles and lower rates of transcytosis relative to peripheral vasculature, and (3) the active transport of molecules between blood and brain [14]. TJs restrict the paracellular diffusion of ions and hydrophilic solutes across the BBB, leading to the high transendothelial electrical resistance (TEER > 1800 Ω.cm^2^) as measured in situ in rats [15].

We previously reported the establishment of a human BBB model by derivation of endothelial cells from human-induced pluripotent stem cells (iPSCs) obtained from healthy donor [16]. In the present work, we developed and validated a functional BBB co-culture models using iPSCs from HD patients reprogrammed to mimic and reproduce some functional properties observed in vivo in patients and animal models. Several dysregulated pathways have been previously reported in in vitro BBB model from HD [12], but an extensive comparison of BBB properties such as transcellular and paracellular transport has not yet been described.

The permeation of molecules across the BBB is regulated by different types of transporters expressed on both the luminal and abluminal membranes, for which their activity depends on transcription factors and nuclear receptors in a tissue- and ligand-specific fashion [17]. Albeit mechanistic studies indicated that the major transport proteins of the ATP-binding cassette (*ABC*) and Solute Carrier (*SLC*) families, categorized in influx and efflux, were functional and correctly polarized, some impairments (e.g., P-gp efflux ratio) were observed for microvascular cells derived from HD iPSCs [7]. HD-patient-derived endothelial cells formed a leak barrier, as indicated by the increased transport of some paracellular markers. The transcriptome analysis provided insights in the dysregulated molecular mechanisms present in the endothelial barrier associated with the HD mutation. Those models may also support further discovery of targeted therapeutic approaches.

## 2. Results

### 2.1. Differentiation of iPSC into Brain Microvascular Endothelial Cells

**Figure 1:**
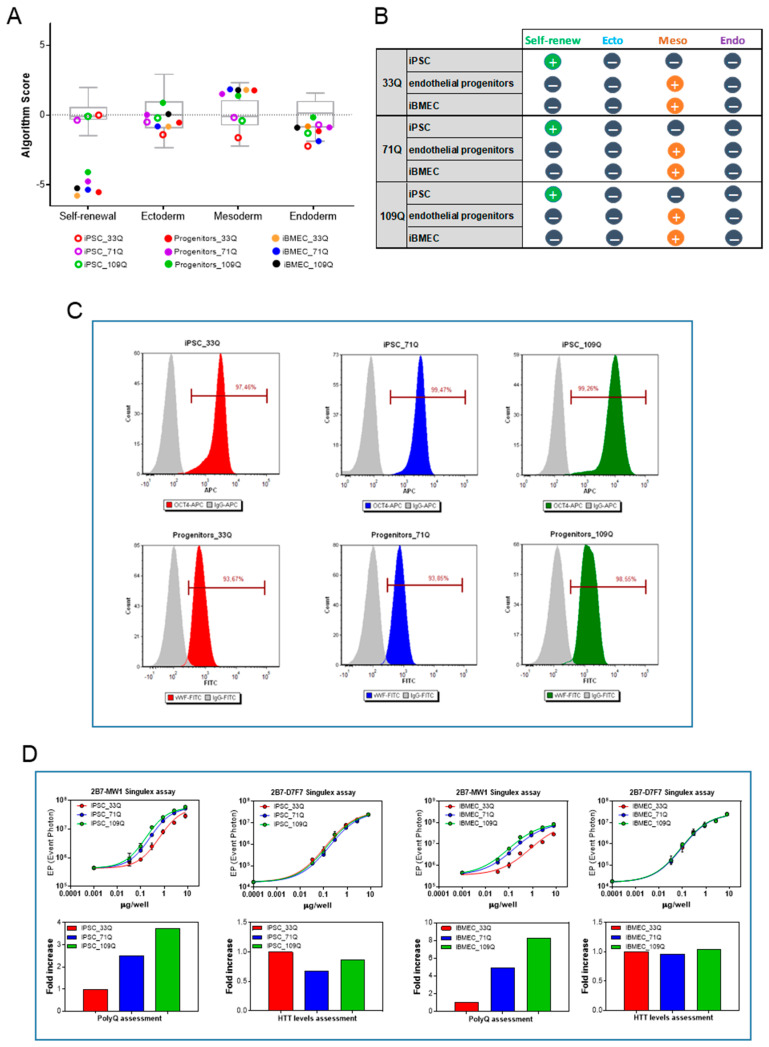
iPSC differentiation into iBMECs. (**A**) Scores box plot. View samples scores (colors) in relation to the range of scores for the undifferentiated reference set (gray) for cultured iPSC (empty circles), progenitors (D8), and iBMECs (D10) (filled circles). (**B**) Summary of gene expression level data in each category for the tree differentiation stages of the three cell lines. (**C**) Representative flow cytometry analysis of OCT4 during routine culture of iPSC and of VWF before final differentiation. (**D**) Analysis of mHTT and HTT expression in 33Q, 71Q, and 109Q iPSCs (left panels) and iBMECs (right panels) using 2B7-MW1 and 2B7-D7F7 Singulex assay, respectively. Curve fittings of the serially diluted samples (described by a four-parameter logistic curve fit) are shown in the top panels, mean ± sd of 3 replicates. The bar charts (bottom panels) reported the fold increase among the different samples (fixing 33Q as reference = 1) and were derived from the EC_50_ of the curve fittings above.

iPSCs differentiation was achieved using previously described protocol [16]; after eight days of mesoderm induction, the mixed population of neuro-endothelial cells was purified by specific adhesion to a collagen/fibronectin matrix. All cell lines, characterized by different CAG repeats length, 33Q (healthy control), 71Q, and 109Q (HD iBMECs), were induced to mesoderm commitment and to the final acquisition of BBB-specific markers, as indicated by the mRNA expression signatures, consistent with the expected directed lineage and phenotypes (Figure 1A,B and Figure S1).

Before using iPSCs as source of brain-like endothelial cells, the cells were characterized for their pluripotency and genomic stability. The expression levels of genes from stem cell markers, and those representative of each of the three germ layers, were assessed by quantitative PCR using pluripotency-score card. iPSCs expressed self-renewal genes and were not committed to any germ layer (Figure 1A,B). The expression of OCT4, analyzed by flow cytometry at the beginning of routine culture, was greater than 95% for all three cell lines (Figure 1C).

The expression of the endothelial marker von Willebrand Factor (vWF) in all iBMECs progenitors confirmed the transition of all iPSCs into cells with an endothelial phenotype (Figure 1C).

The presence of a CAG expansion in the *HTT* gene did not interfere with the proper acquisition of the meso-endodermal fate, and the differences in the levels of expression were not observed (Figure 1D).

To further confirm that the CAG expansion was detectable all along the differentiation process, the Singulex assay employing 2B7 as capture antibody and MW1 as the detection antibodies was used. Indeed, this antibody pair is suitable for revealing the HTT protein in a polyQ-dependent fashion, as the MW1 antibody displays an apparently higher affinity for HTT bearing expanded polyQ repeats. On the other hand, a polyQ-independent Singulex assay employing the same capture antibody (2B7) and D7F7 (~aa1220) as the detection antibody was used for interrogating the expression levels of HTT protein (Fodale et al., submitted to Journal of Huntington’s Disease). As it can be observed in Figure 1D, the 2B7-MW1 antibody pair was able to properly discriminate the increase in polyQ expansion (33Q, 71Q, and 109Q) in both iPSCs and iBMECs, while no modulation in HTT levels imparted by the different CAG repeats has been detected by the 2B7-D7F7 Singulex assay. All iPSC lines consistently produced comparable amounts of endothelial progenitors across a total of more than 10 independent cycles of differentiation and multiple users. From one million iPS cells, we obtained about 5 million endothelial progenitors for both the control and HD iBMECs, indicating a successful differentiation outcome.

**Figure 2:**
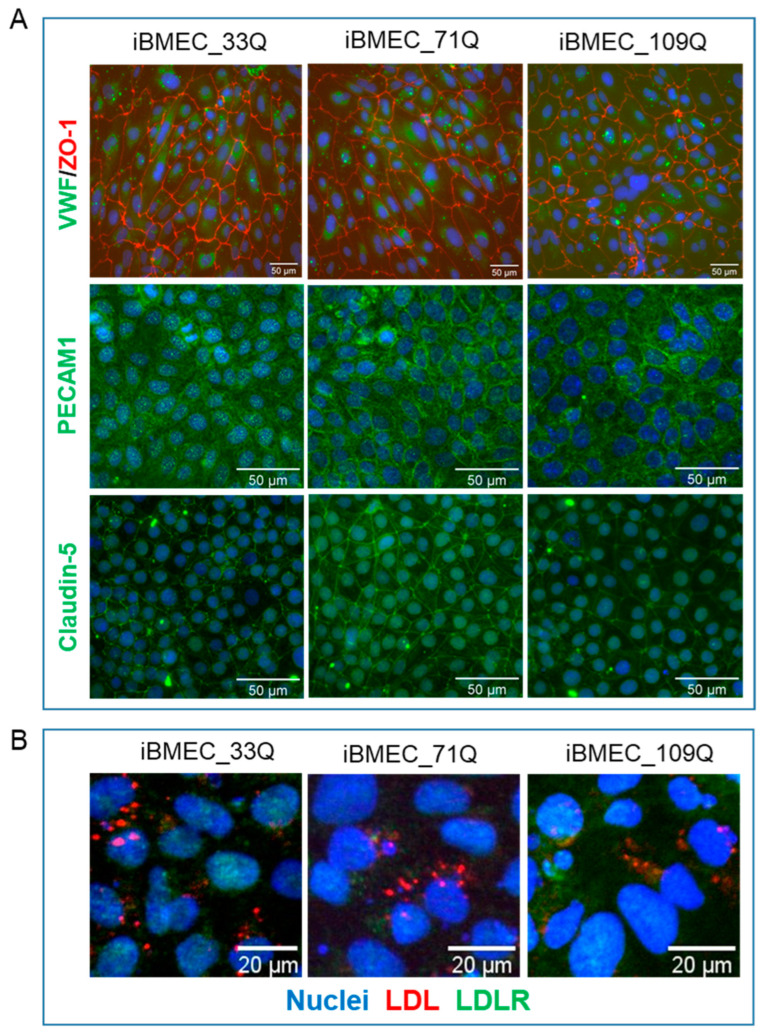
iPSC differentiation into iBMECs. (**A**). Representative immunofluorescence staining, at d1 co-culture in transwell filters, demonstrating the expression of endothelial relevant proteins: vWF, ZO-1, PECAM1, and Claudin-5. Nuclei were counterstained with Hoechst (blue). Scale bar represented 50 µm. (**B**) LDL uptake (in red) by iBMECs (at D10). In green, the intracellular distribution of LDLR was also reported (scale bar indicates 20 µm).

Following the final purification on plates coated with collagen and fibronectin, all cell lines expressed the endothelial markers Von Willebrand Factor (vWF), CD31 (PECAM-1), and the tight junction’s proteins Claudin-5 and ZO-1 (Figure 2A). All the three iBMECs expressed vWF at comparable levels as well as PECAM-1 at D10 (day 1 in co-culture). Claudin-5 and ZO-1 showed a distinct localization to cell borders, similarly for all iBMECs. We did not observe the difference in the Claudin-5 cellular localization as reported by Lim et al. [12] for the HD cell lines. A degree of diffuse expression pattern within the cytoplasm was also observed, consistent with the recent evidence of non-tight junctions and nuclear roles for these proteins [18].

Differentiated endothelial cells also expressed the LDLR and were able to internalize LDL particles (Figure 2B). The red fluorescence observed in cells indicated that LDL can be efficiently transported into the cells and accumulate intracellularly. The uptake of LDL particles into primary endothelial cells specifically by LDLR was shown by the colocalization of fluorescence-labelled LDL particles and LDLR.

### 2.2. Comparison of Barrier Properties of Healthy and HD Models

**Figure 3:**
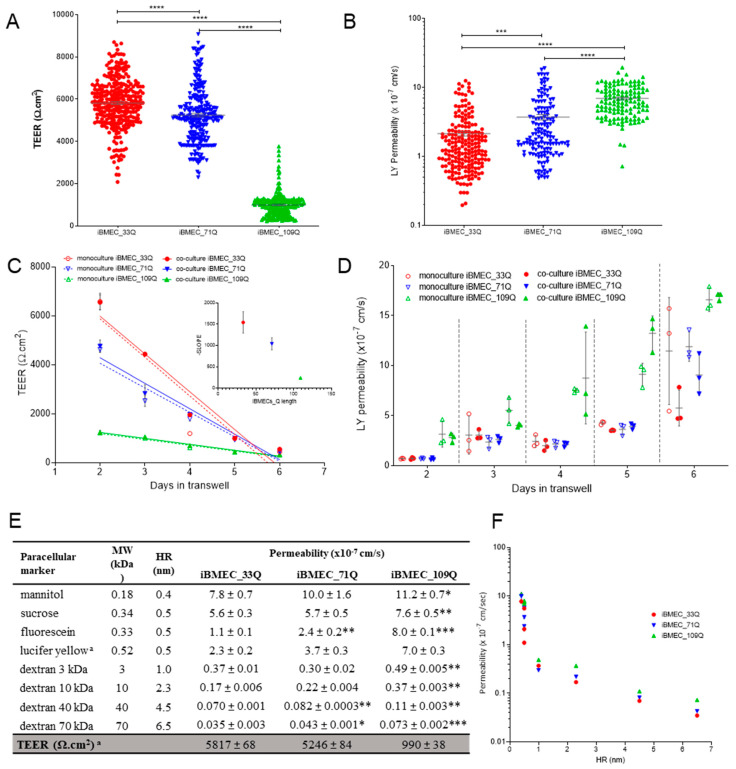
Barrier properties. (**A**) TEER measurements and (**B**) LY permeability at day 1 co-culture of independent samples, *n* > 100. Statistical analysis: two-way ANOVA followed by Bonferroni post hoc test, where *** *p* < 0.001 and **** *p* < 0.0001. (**C**) TEER as a function of time in mono- and co-culture with astrocytes and (**D**) corresponding LY permeability time-course in mono- and co-culture, *n* = 3. (**E**) Permeability in the BBB models of paracellular markers with different molecular weights (MW) and hydrodynamic radius (HR). Results are mean ± sd with *n* > 6 from at least two separated experiments. Statistical significance was analyzed by Student’s *t*-test against iBMEC_33Q: * *p* < 0.05; ** *p* < 0.01 and *** *p* < 0.001. ^a^ Statistics showed above (**A**,**B**). (**F**) Relationship of permeability and HR (nm) for paracellular markers listed in (**E**).

The integrity of the cell monolayer was assessed by trans-endothelial electrical resistance (TEER). At day 2 post-seeding on transwells, all cell models exhibited an increase in TEER (Figure 3A). However, iBMEC_109Q showed more than 5-fold lower TEER than iBMECs 33Q and 71Q, but it was still close to the values thought to be physiological [19]. The average measured TEER values across iBMECs 33Q and 71Q (>5000 Ω·cm^2^) reflected previously published values [16]. By contrast, iBMEC_109Q formed a leakier barrier (ca 1000 Ω·cm^2^). However, an increase in the paracellular transport of LY, used as probe, was observed (Figure 3B) from the healthy model to both HD models.

A previous report [12] indicated that endothelial cells derived from both 71Q and 109Q iPSCs had a very low TEER (<400 Ω·cm^2^) at 72 h post-seeding. To understand if the earlier time-point might be responsible for this difference, we compared the TEER trend for additional 4 days in co-culture. TEER values were maintained for at least two additional days and were not dependent on the presence of *human* astrocytes, as supportive cells, at the bottom of the basolateral chamber (Figure 3C). For iBMECs 33Q and 71Q, the time-courses were similar with a peak at day 1 in the transwell, and then there was a decrease until day 5. A much less steep decrease was observed for iBMEC_109Q since the peak was much lower, although it reached the same time point. When considering the TEER decrease as a linear function of time, the slopes plotted against the CAG lengths indicated that the barrier-forming capacity was impaired in the HD models. The increase in paracellular permeability of LY during this time course for all conditions reflected the observed TEER changes (Figure 3D).

We next compared the permeability of different compounds known to be transported by the paracellular route (Figure 3E) chosen based on their size and charge. All cell models discriminated them in a size-dependent manner with an inverse correlation with both the molecular weight and the hydrodynamic radius (Figure 3F).

Permeability values obtained with small hydrophilic compounds such as LY and tracers with higher molecular weight and hydrodynamic ratio were comparable to the ones obtained in a 3D self-organized microvascular model of the human BBB, with endothelial cells either in monoculture or in tri-culture with pericytes and astrocytes [15].

In addition, they were also close to those measured in vivo in rat cerebral microcirculation, with a P = 1 − 2 × 10^−7^ cm/s for LY [20], a P = 3.1 ± 1.3 × 10^−7^ cm/s for a 10 kDa FITC-dextran [21], and P = 1.37 ± 0.26 × 10^−7^ cm/s for a 40 kDa FITC-dextran [22].

However, the iBMEC_109Qs were leakier than iBMEC_33Q for all dextrans, whereas iBMEC_71Qs were significantly leakier only for the bigger dextrans tested, 40 kDa and 70 kDa (Figure 3E). This variation in the permeability of dextran-FITC molecules is in agreement with the observed increase in a HD BBB-Chip model [23]. As reported in Figure 3E, there were no significant differences in the permeability of non-charged molecules such as sucrose and mannitol between the iBMECs 33Q and 71Q, whereas the permeability coefficients were slightly higher only in iBMEC_109Q. In a different manner, the permeability of the anionic low molecular weight markers, fluorescein, and LY increased in the HD models. This was also confirmed at longer time-points (Figure 3D) for LY, for which its permeability exceeded 5 × 10^−7^ cm/s at day 4 for iBMEC_109Q, while for iBMECs 33Q and 71Q, it waited until day 5 to reach this value.

**Figure 4:**
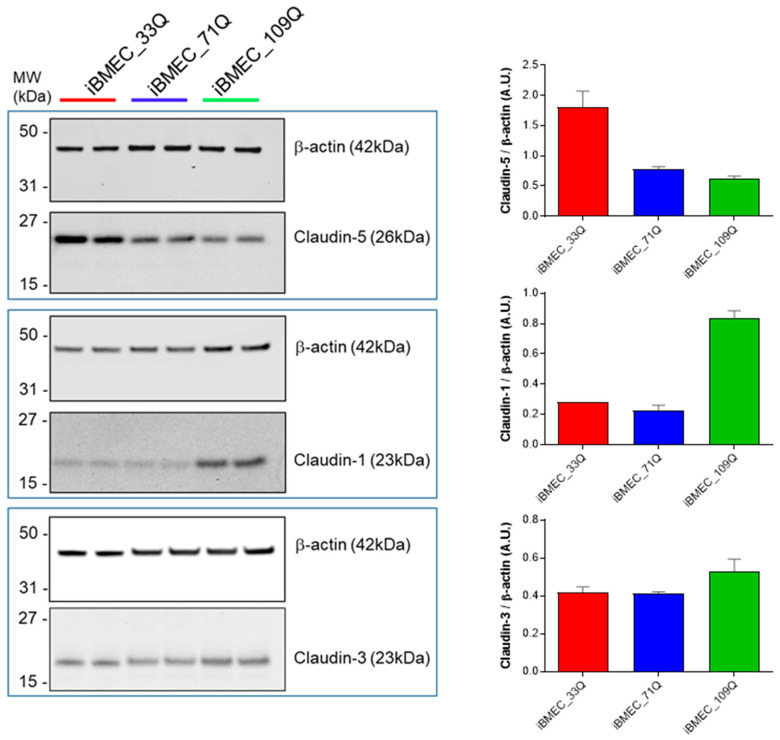
Claudins. Representative cropped Western blot confirming expression of Claudins 5, 1, and 3 in iBMECs. Β-actin was used as the loading control, and relative expressions are reported in the neighbouring bar charts.

A critical protein implicated in ensuring tight junctions’ formation in brain endothelial cells is Claudin-5. To explore the possibility that there is a loss of this regulation in the HD models, at day 1 co-culture, iBMECs (33Q, 71Q and 109Q) were collected and protein expression levels of the selected claudins were analyzed by Western blot analysis (Figure 4). We found Claudin-5 downregulation in HD models; on the contrary, upregulation of Claudin-1 was observed only in iBMEC_109Q. Claudin-1, although rarely expressed at the normal blood–brain barrier (BBB), has been reported to be upregulated in pathological conditions and associated with an endothelial proinflammatory phenotype. The analysis of post-stroke *human* and *mouse* blood microvessels indicated that Claudin-1 was highly expressed in leaky brain microvessels and there was a corresponding decrease in Claudin-5 expression [24]. The significantly decreased expression of Claudin-5 has also been observed in the post mortem analysis of the brain–blood vessels of HD patients and was found to be associated with increased permeability as well [5]. We also analyzed the protein levels of Claudin-3, reported to be specifically expressed at high levels in the cerebral endothelium and to have a barrier function [25], but the levels were comparable among the BBB models.

These results suggested that the increased leakage to paracellular markers of the HD models could be attributed, at least in part, to an imbalance in claudins expression such as Claudin-5 and Claudin-1.

### 2.3. Transcriptional Profiling of Brain-Like Endothelial Cells

**Figure 5:**
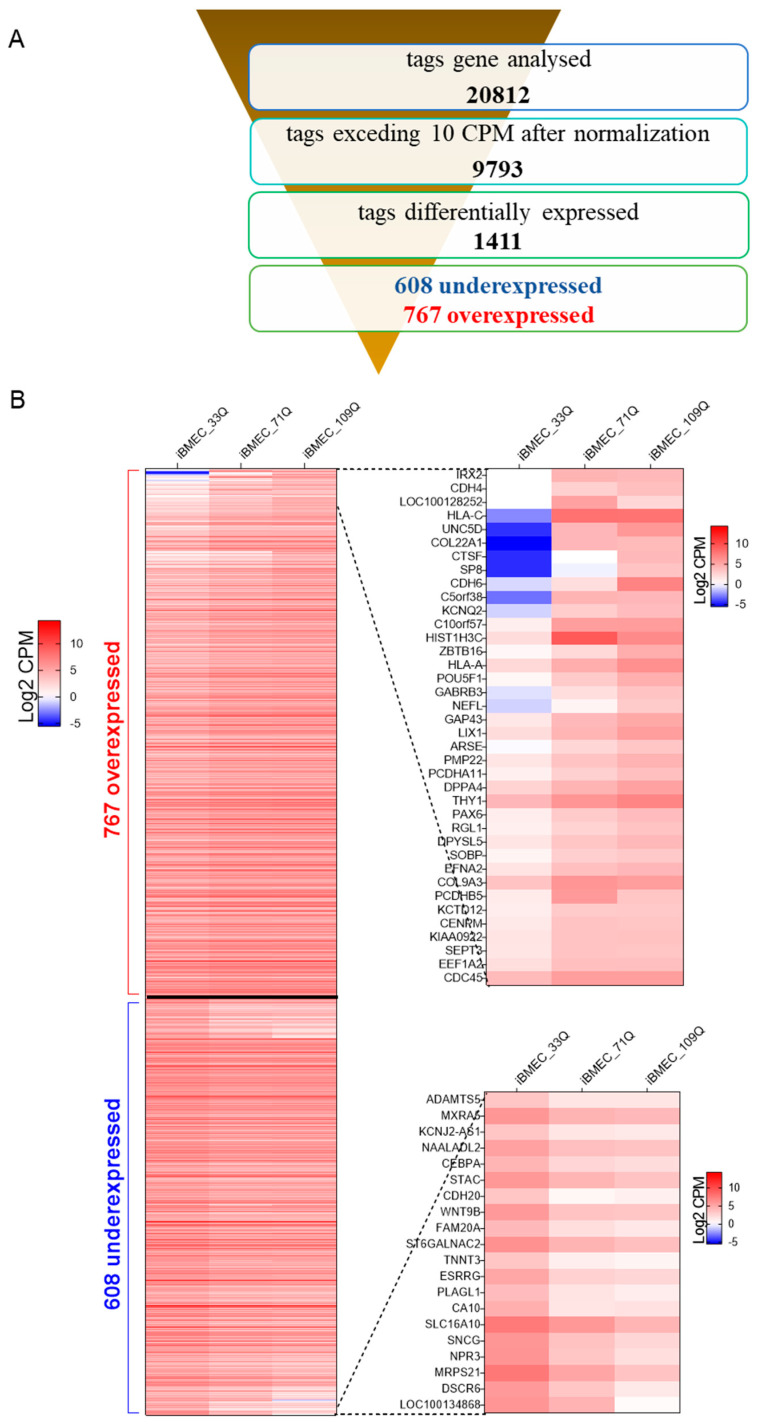
RNA seq. (**A**) Overview of RNA-seq of the samples. The output value for the detected expression is the normalized counts per million (CPM). (**B**) (Left) Heat map (showed as Log2 of CPM, mean of three experimental replicates) of tagged differentially expressed genes, when both HD-iBMECs are overexpressed or underexpressed vs. healthy-iBMEC with statistical significance determined by Student’s *t*-test of *p* < 0.05. (Right) The results of the top up- and downregulated genes are shown in magnification, where the fold change of both HD-iBMECs vs. healthy was more than 3-times increased or decreased.

Transcriptional dysregulation is a central feature of HD [26] and has been demonstrated in a wide range of tissues in both HD patients and animal models [27]. Although the primarily affected brain region in HD is the striatum [28], CNS degeneration can also be associated with dysfunction at the cerebrovascular level [29]. Our RNA-seq results revealed that about 1400 genes were differentially expressed between healthy and the HD models, with more than 700 genes significantly (statistical difference using Student’s *t*-test set at *p* < 0.05) upregulated and about 600 genes downregulated in HD (Figure 5A,B). Differentially expressed genes between HD-iBMECs and healthy iBMEC, where the fold change of both HD-iBMECs vs. healthy was more than three-times increased (38) or decreased (20), are shown in Figure 5B (right) or Table S5.

Several factors contribute to the physical barrier of BBB and are responsible for the formation and maintenance of the endothelial structural lining including adherens junction (AJ) and tight junction (TJ) proteins. The major junctional molecules of the adherens junctions such as E, P, and *N*-cadherin and VE-cadherin, important in cell–cell adhesion through homotypic interaction, were expressed in iBMECs. VE-cadherin, encoded by *CDH5*, was significantly downregulated in the HD model derived from the 109Q cell line. The low levels of *CDH5* are indicative of increased barrier permeability, a method exploited by some viruses [30] in agreement with its role of stabilization of blood vessel assembly. The significant upregulation of *CDH4* (cadherin 4), *CDH6* (K-cadherin), and *CDH11* (cadherin 11) was observed on both HD models (Tables S1 and S2).

The three principal families of tight junction proteins, reported to be an important regulator of TJ assembly and functions such as Claudins, Occludin, ZO-1, ZO-2, Marvel/D, and Ig-like junctional adhesion molecules (JAMs), were also present (Table S2). Among them, the downregulation of *Marvel/D2* (tricellulin) and *D3* and the upregulation of mRNA of *JAM3* and Claudin-1 (CLDN1) were found in the HD models, the latter in agreement with the higher expression found at the protein levels (Figure 4). Three additional members of the tricellular TJ family were also present: angulin family angulin-1 (*LSR*), angulin-2 (*ILDR1*), and angulin-3 (*ILDR2*). In the BBB, angulin-1 plays an important role to constitute the functional TJ barrier in conjunction with tricellulin [31,32].

The transport systems of the *SLC* and *ABC* families play a central role in the molecular trafficking of nutrients and drugs through BBB. Among the 45 *ABC* genes categorized into seven families (*ABC-A*, -*B*, -*C*, -*D*, -*E*, -*F*, and -*G*) tagged in the transcriptome, only 3 were not detected in any cell line. A significant 2-fold upregulation was observed for *ABCA2* and *A7*, for which their variants have been associated with AD and were identified as dysregulated in another study [12]. The strongest downregulation was observed for *ABCA12* and *A9* (4.2- and 3.5-fold, respectively) (Table S2).

The upregulation of *ABCB1* (Pgp) in the HD model was not observed in contrast with previous reports [12]. These data were also in agreement with the comparable functional activity of the transporter measured in bidirectional transport studies in our models, using prototypical Pgp substrates (Table 1 and Table 2).

Within the C family (*ABCC*), several genes of multidrug-resistance-associated proteins were expressed corresponding to MRP1, 2, 3, 4, 5, 6, and 7, with upregulation in the HD cell lines of *ABCC4* (MRP4, 25 FC) and *ABCC6* (MRP6, 7 FC), as observed [12].

*ABCG2* (BCRP) was the transporter with the highest expression of all *ABC* members. Recent proteomic analyses indicated that BCRP is the most abundant transporter at *human* BBB, with approximately 2-fold higher expression in humans than in *rodents,* which could imply a more prominent role of BCRP in brain penetration in humans [33].

The *SLC* group is the largest family after the G protein-coupled receptor (GPCR) superfamily, counting over 400 members organized into 66 families. Unlike primary active transport such as *ABC* transporters, *SLCs* function by facilitative diffusion and secondary active transport and they are mainly bidirectional. To date, a total of 287 *SLC* genes have been identified in the brain, and those expressed in the endothelial cells of the BBB contribute to keeping the brain isolated from toxic substances and mediate the transport of a wide range of essential nutrients and metabolites [34].

We observed the expression of members belonging to all families in both the healthy and HD models. The known transporters of energy metabolites (e.g., *SLC2A*, *SLC16A* families), amino acids, neurotransmitters (*SLC1A*, *SLC7A*, and *SLC38A* families), ions (zinc transporter family *SLC39A*), organic anions (*SLCO* family), and organic cations (*SLC22A* family) are expressed (Table S2).

The analysis of the transcriptomic data indicated that the glucose transporters *SLC2A3* (GLUT3) and *SLC2A1* (GLUT1); the Na (+)-dependent multivitamin transporter *SLC5A6* (SMVT); the amino acid transporter *SLC7A5* (LAT1); the monocarboxylate transporter *SLC16A1* (MCT1); mitochondrial carriers such as *SLC25A3* (MPCP), *SLC25A5* (ANT2), and *SLC25A6* (ANT3); and the choline transporter *SLC44A2* (CTL2) were the most abundantly expressed (Table S3).

Glucose transporters (GLUTs) at the blood–brain barrier maintain continuous high glucose and energy demands of the brain. The sodium-independent facilitating transporters *GLUT1* and *GLUT3*, identified as major glucose transporters [35], were present in all models with higher expression, as a confirmation of a physiological phenotype of the endothelial cells.

In agreement with previous works that reported the expression in endothelial cells isolated from brain capillary [36], genes from *SLC1A* family coding for EAAT-1 (*SLC1A3*), EAAT-2 (*SLC1A2*), EAAT-3 (*SLC1A1*), and EAAT-4 (*SLC1A6*) were expressed. EAAT-4 was downregulated in the HD models. EAATs are involved in the efflux of glutamate across the BBB and ensured low levels of this neurotransmitter in interstitial brain fluids. The two members of the ASC system (Transport of Large and Small Neutral AAs), ASCT1 (*SLC1A4*) and ASCT2 (*SLC1A5*), that have been described in different BBB models [37] were also present.

Solute carriers for organic anions and cations of the *SLC21/SLCO* and *SLC22* families accept a broad range of cationic and anionic compounds as substrates, including environmental pollutants and various drugs such as antibiotics and nucleosidic antiviral drugs, non-steroidal anti-inflammatory agents, and some antiepileptic drugs [38]. Members of *SLCO* can trigger the blood-to-brain transport of opioid analgesics, such as deltorphin II and DPDPE ([D-penicillamine (2,5)]-enkephalin), and are potential targets for the treatment of pain and cerebral hypoxia [34]. *SLCO1A2, SLCO2A1, SLCO3A1, SLCO4A1, SLCO4C1*, and *SLCO5A1* were expressed in all models. *SLCO1A2* (OATP2), one of the organic anions transporting polypeptides (OATPs), is a transporter for many drugs, including statins, morphine derivates, and antibiotics, and it has been reported to be expressed in *human* brain microvessels and brain capillary endothelial cells and absent in *mouse* microvessels [39]. While *SLCO2A1* (OATP2A1) was downregulated, the *SLCO5A1* (OATP5A1) isoform was upregulated in the HD model. The latter is an orphan OATP transporter, but its role in the cellular uptake of drugs has not been characterized so far.

Many members of the mitochondrial carrier family (*SLC25*), the largest of the transporter families, were also expressed. They transport a variety of solutes such as ATP, ADP, phosphate, tricarboxylic acid cycle intermediates, cofactors, amino acids, and carnitine esters of fatty acids [40]. The most abundant in all models was *SLC25A3* (the mitochondrial phosphate carrier PiC). Dysfunctional *SLC25* proteins are involved in pathological conditions [41]. In the HD model, *SLC25A8* (UCP2) and *SLC25A48* were downregulated (2–3 fold) and significant (about 2 fold) upregulation was observed in the HD model for *SLCA13* (aspartate/glutamate carrier 2) and for *SLC25A32* (mitochondrial folate transporter).

In the monocarboxylate transporter family, *SLC16A1* (MCT1) exhibited the highest expression levels, followed by *SLC16A10* (MCT10), which was particularly downregulated and its expression fell to 10% in a CAG-dependent manner. The endothelial cells of the blood vessels in the brain have been reported to express MCT1, which probably mediates the transport of lactate and ketone bodies across BBB [42]. MCT10 is an aromatic amino acid transporter and is also known as T-type amino acid transporter1 (TAT1).

The levels of members of the *SLC15* family, reported to be involved in peptide transport across the epithelial layer in animal organs and across the BBB [43], increased in the HD models, specifically *SLC15A1, A3*, and *A4*. In particular, significant (2–3 fold) upregulation was observed for *SLC15A4* (peptide/histidine transporters PhT1). Although the peptide-histidine transporters PhT1 and PhT2 (*SLC15A3*) are present in the brain, their functional importance is unknown, and the expression of PhT1 transcripts was reported to be significantly upregulated in inflamed areas of the colon of patients with Crohn’s disease and ulcerative colitis [44].

The transcriptomic map of genes encoding RMT receptors showed comparable levels of major BBB receptors. However, significant upregulation in the HD models of the HDL receptors scavenger receptor class B type I (*SCARB1*) was observed. This receptor has been proposed to have a role in neuroinflammation, neurovascular dysfunction, and subsequent neurodegeneration [45], whilst the downregulation of *LRP10* was observed in the HD models. Mutations in the LRP10 (low-density lipoprotein receptor-related protein 10) gene have been identified recently in individuals affected by PDand dementia with Lewy bodies [46].

The BBB models also expressed a variety of phase I and phase II enzymes and regulators of brain functions. The glycolytic enzymes of the enolase family such as *ENO1*, *ENO2* and *ENO3* were statistically significant upregulated in HD models, in agreement with studies conducted by others groups [47,48].

We observed an upregulation in the HD models of several genes involved in glucose metabolism, including the pyruvate dehydrogenase complex (PDHC) and the tricarboxylic acid (TCA) cycle (Table S2). Our findings, although not always statistical significant (see Table S2), agreed with imbalanced enzymatic activities in the Q175 cortex [49], particularly for succinate dehydrogenase (*SDHB* and *C*), PDHC complex, aconitase (*ACO1* about 2 fold), succinyl thiokinase (*SUCLA2* about 4 fold), and isocitrate dehydrogenase (*IDH1, 2, 3A*, and *3B*), which suggested an upregulation of the TCA cycle. We also found increased expression in HD model of citrate synthase (*CS*) and malate dehydrogenase (*MDH*).

The expression of alkaline phosphatase (*ALPL*) increased in the HD model (about 2-fold), while the levels of γ-glutamyl transpeptidase (*GGT1*) and detoxification enzymes such as *CYP1A1* and *GSTO1* decreased in the diseased models.

Higher levels of phospholipase-C γ1 (*PLCG1*) were found in HD models. PLCG1 has been proposed as a mediating factor in schizophrenia, bipolar disorder, Alzheimer’s, Huntington’s, and epilepsy [50].

The HD models showed the upregulation of some RNAs involved in the innate immunity (C-X-C motif chemokine 12 (*CXCL12*)—a chemokine protein) (2–4 fold) and hypoxia response genes (*LDHA, ALDOA*) (about 2-fold). Brain endothelial cells link peripheral immune responses to the CNS by acting as the sensors and mediators of immune processes in the periphery [51].

We found a significant upregulation in the HD models of the RNA for *HLA-A and HLA-C* (>6 fold), and this correlated with the upregulated expression found in degenerative and/or inflammatory neurological disorders [52]. Both were present also in the healthy model, but the relative transcript abundance was very low and, consequently, attained high significance.

### 2.4. Functionality of Different Transport Mechanisms

**Figure 6:**
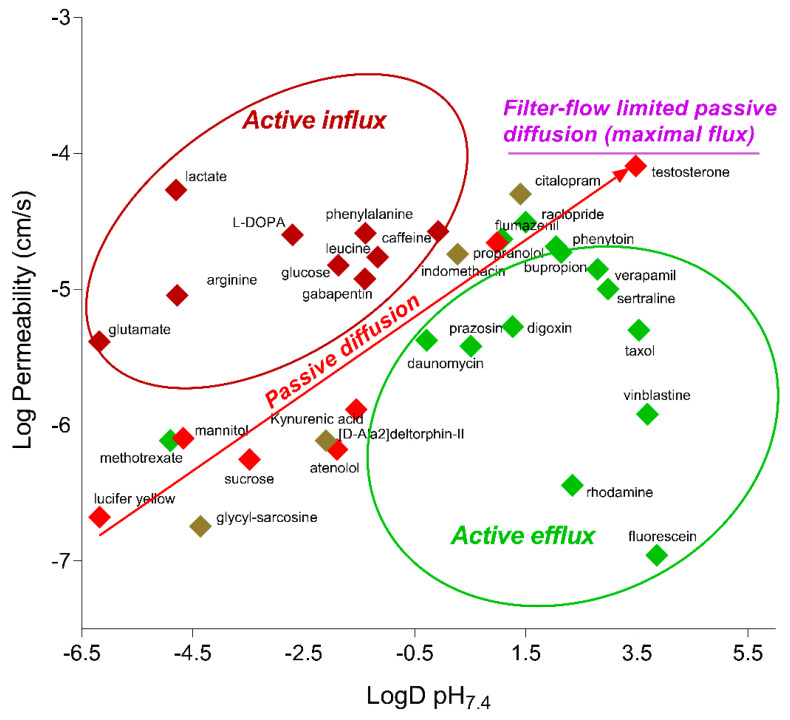
The Log Permeability values for tested compounds in iBMEC_33Q, plotted against their corresponding Log D pH_7.4_ values.

To characterize the functional BBB properties of the HD models in comparison with the iBMEC_33Q, the transport of small molecules with different transport mechanisms was evaluated. As reported in Table 1, most of the tested compounds were transported at comparable rate in all models, including those subjected to influx and efflux. The correlation with the physicochemical properties such as the Log D at pH 7.4 (Figure 6) indicated that compounds known to be substrates for influx transporters (such as amino acid transporters) had higher permeability values than expected from their Log D value, while compounds known to be substrates for efflux transporters (Pgp, MRP, and BCRP) had lower permeability values than expected. These data suggested that major Adenosine Triphosphate (ATP), *ABC*, and *SLC* transporters were functional and polarized.

Polarized transport is reported in Table 2. Efflux transporters of the *ABC* family such as *ABCB1* (Pgp) and *ABCG2* (BCRP) were asymmetrically distributed as indicated by the higher transport of vinblastine and prazosin from the abluminal to luminal side, respectively. The co-administration of elacridar and KO143, respectively, Pgp and BCRP inhibitors caused a significant reduction in the efflux ratio. In addition, the antiepileptic drug phenytoin, the substrate of MRPs and the antidepressant citalopram, and the substrate of Pgp were transported more efficiently in the HD models (see Table 1).

*SLCs* expressed in the endothelial cells of the BBB contribute to keeping the brain isolated from toxic substances and are necessary for absorbing essential components from the blood. The functionality of *SLCs* with higher mRNA expression was chosen for further characterization. Glucose (transporter GLUT1 also known as *SLC2A1*), phenylalanine and leucine (transporter LAT1 also known as *SLC7A5*), and lactate (transporter MCT-1 also know as *SLC16A1*) [53] were transported at high rates and had a comparable value among the models (see Table 1). Glucose transport was inhibited by the specific inhibitor phloretin by nearly 50–60% in both directions (apical to basal and basal to apical) for all iBMECs (Table 2). *GLUT-1* was found at particularly high levels in endothelial cells and in the epithelial-like barriers of the brain, and it has been reported to be was expressed in both the luminal and abluminal membranes [54].

The transport of leucine was also reduced by about 80% in the presence of JPH203, a selective LAT1 inhibitor, suggesting that LAT1 is the main functional Na^+^-independent leucine transporter in all models.

Consistent with the presence in the brain endothelial cells of transporters that can mobilize glutamate from the CNS parenchyma to the luminal zone of the blood stream through mechanisms that are not well understood [55], a polarized brain-to-blood transport of glutamate was quantified. However, the luminal and, to a lesser extent, abluminal glutamate uptake were higher in iBMECs_109Q, with a resultant decrease in the efflux ratio (Table 2). Several members of *SLC1A* family were expressed in all models (Table S2) with *SLC1A6* (EAAT-4) and *SLC1A5* significantly dysregulated in the 109Q model (down and up, respectively). Such differential expression could be the reason for perturbations in the glutamate/GABA-glutamine cycle and may increase glutamate burden through decreased efflux and/or increased influx across the BBB.

The permeabilities of drugs could involve multiple processes (passive, influx, and efflux) and substrates can be transported by a combination of carrier-mediated mechanisms, possibly working in opposite directions. While the ABC transporter family consists of unidirectional transporters that allow the exit of substrates from the luminal surface of the cell into the blood, SLC transporters are mainly bidirectional [56]. The functional interplays of ABC and SLC transporters in regulating drug transport across the BBB can be reflected by the observed efflux ratio close to one of verapamil. Verapamil has been reported to be a substrate of Pgp (*ABCB1*) and OCTN1 and 2 (SLC22A1 and A5, respectively). *SLC22A5* has been identified as the carnitine transporter from the blood to the brain, and its expression at the RNA level (Table S2) might explain the comparable permeability from the apical to basal and from the basal to apical (Table 2) of verapamil.

The antidepressant drug citalopram showed higher permeability in the HD models (Table 1), which could be due to the imbalance of different mechanisms. The citalopram is reported to be a substrate of MRP1 (*ABCC1*), and a single nucleotide polymorphism (SNP) in this gene was found to be significantly associated with citalopram response in patients [57]. We observed the downregulation of this gene in HD models, albeit with low significance (Table S2).

The dynamic range of all models determined between the highest (testosterone) and lowest (mannitol) permeability values was about 80–100, indicating that they were able to discriminate among drugs that cross the BBB by different mechanisms.

### 2.5. Receptor-Mediated Transport Mechanisms

**Figure 7:**
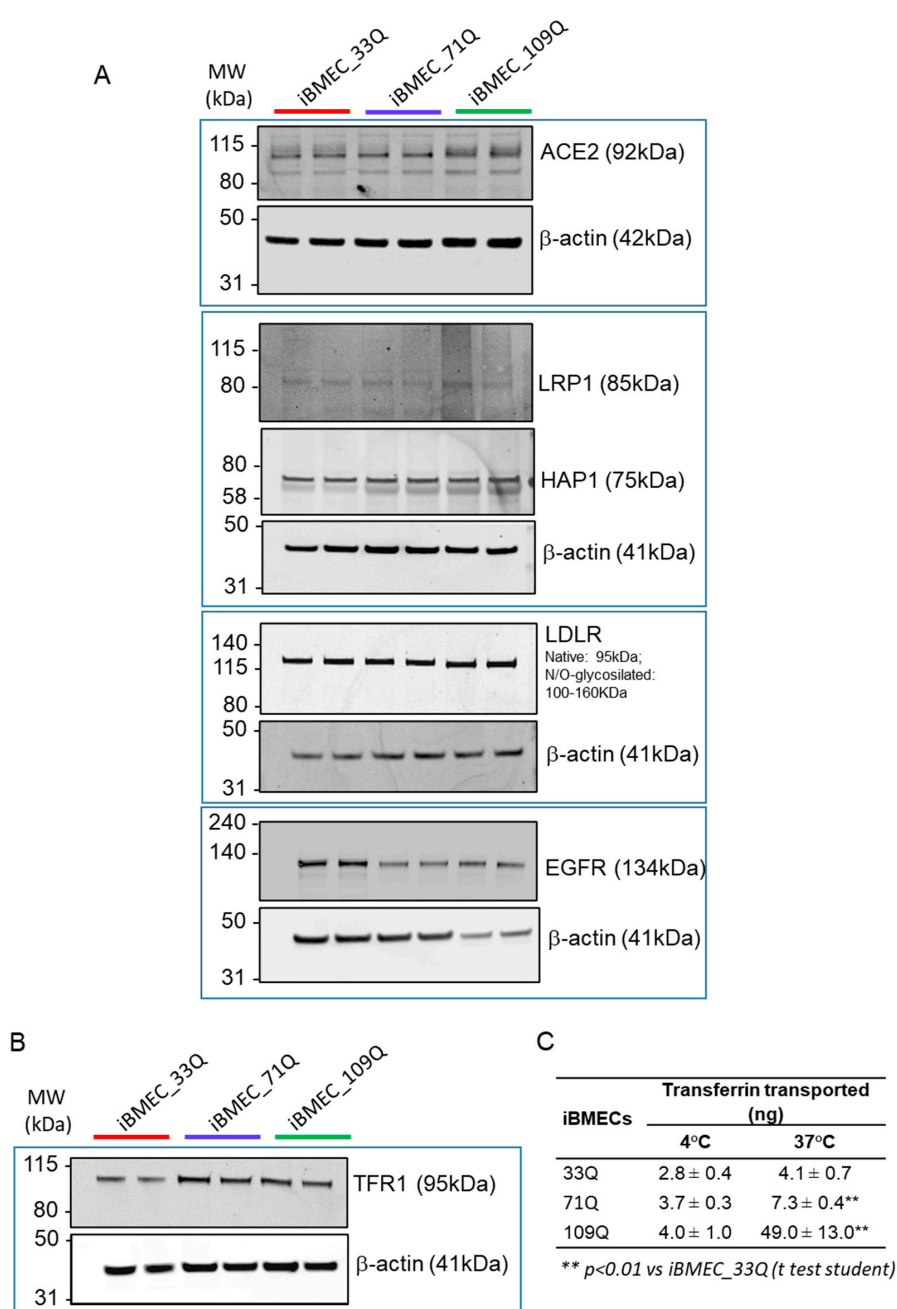
Expression levels of major BBB receptors and receptor-mediated transcytosis of Transferrin in the iBMECs. (**A**) Representatively cropped Western blot confirming the expression of ACE2, LRP1, HAP1, LDLR, and EGFR in iBMECs. β-actin was used as the loading control. (**B**) Representative cropped Western blot confirming the expression of TFR1 in iBMECs. Β-actin was used as the loading control. (**C**) iBMECs support the transcytosis of the transferrin; measured at 37 and 4 °C in the basal compartment after 2 h is the mean ± sd.

The transport of large molecules has been described to be very low in the healthy brain. This specialized mechanism is due to the presence of specific receptors that are able to mediate endocytosis after ligand binding [58]. Major receptors, such as the transferrin receptor (TFRC/TFR1), the lipid transporters low-density lipoprotein receptor (LDLR), and LDLR-related protein 1 (LRP1), were expressed in both healthy and HD models (Figure 7A,B). The putative SARS-CoV-2 receptor ACE-2 was also expressed [59].

We previously reported the transport of an antibody against TfR receptor, for which its uptake was about 0.2% of dose due to the restrictive nature of the model [16]. Here we investigated the permeability of a fluorescent-labelled Transferrin. We also addressed whether any transport differences exist between 37 °C and 4 °C. The analysis of basal accumulation revealed a temperature-dependent transport of transferrin (Figure 7C). However, both iBMEC_109Q and iBMEC_71Q showed higher levels of accumulation (about 5 and 2 fold, respectively), despite the fact that the protein levels of the receptor were comparable (Figure 7B). The paracellular leakiness of the HD BBB models (Figure 3E) could explain the higher rate of transcytosis observed, enabling the passage of much larger molecules. The principal determinants of large molecule flux through TJs have been reported to be ZO-1, Occludin, and members of tricellulin family such as MarveD1, D2, and D3. Based on the RNA-seq analysis, we found a downregulation of both D1 and D2 isoforms in the HD models, likely resulting in the loss of control of flux modulation of larger molecules, which is also in agreement with the higher permeability of fluorescent-tagged dextrans with increased molecular weight.

On the other hand, the dysregulation in the transcytosis of the HD models could also be due to differential intracellular sorting of the ligand after binding to its specific receptor. Both the internalization pathway and the mechanisms downstream the internalization can sort the ligand–receptor complex for different destinations. A recent work described that differential sorting is regulated by intracellular tubules [60] or tubular networks and the chains of vesiculo-vacuolar organelles (VVOs).

The formation of tubules from endosomes is regulated by multiple effector proteins and parallel pathways, which have not been fully characterized. Rab GTPases, which act upstream of effector proteins, are considered the master regulators of sorting. Villasenor and coauthors [60] found that the constructs sorted for degradation to lysosomes exhibited impaired transport along such tubules and that the overexpression of adapters, such as Rab17, induced dFab transcytosis across a BEC monolayer in vitro. In the kinetic transport studies of transferrin, they found a co-localization of Rab17 with Tf-positive tubules.

In our HD models, while we found a comparable level of *Rab17* among the models, a downregulation of *Rab31* was observed (Table S2). Rab31 has been reported to have a major role in the degradative trafficking pathway of ligand-bound EGFR [61]. Higher rate of transferrin transcytosis in the diseased models could be explained by the lower levels of Rab31 that channeled less ligand-bound receptors to the degradative pathway. The complex can either be recycled back to the luminal side or transcytosed to the abluminal side, leading to increase basolateral (brain) accumulation, after dissociation from the receptor.

### 2.6. In Vitro–In Vivo Correlation

The main application for these models would be permeability assessment rather that disease modelling, and for that reason, we compared the in vitro measured permeability of selected therapeutics with the data from the clinic as the in vivo concentration of unbound drug in *human cerebrospinal fluid* (CSF, K_p_,_uu,CSF_).

**Figure 8:**
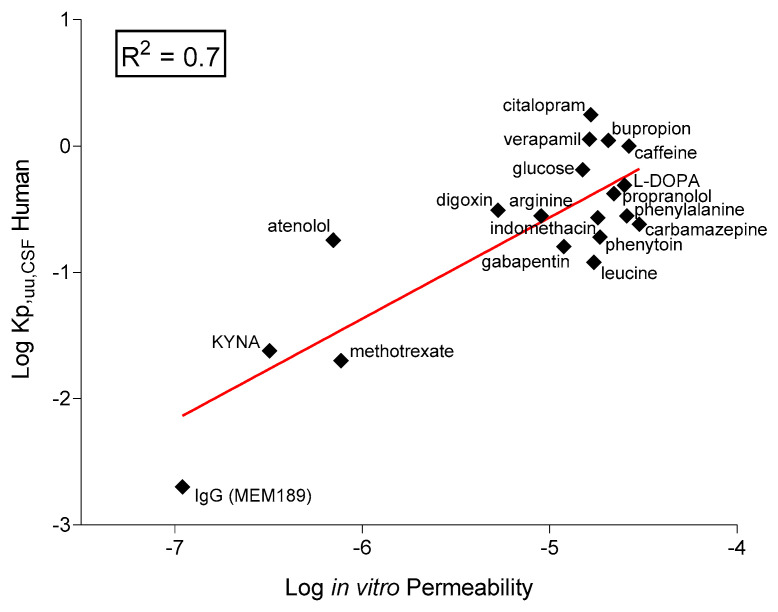
In vitro–in vivo correlation. Correlation between in vitro log permeability from iBMEC-healthy and in vivo *human* Log K_p,uu,CSF_ (data collected from the literature, Table S4). The solid line is the linear regression with an R^2^ value of 0.7.

This analysis included small hydrophilic compounds such as atenolol and known CNS-permeable and impermeable drugs and antibodies (Figure 8). Using the data obtained in the 33Q model, we found a good correlation (R^2^ = 0.7) in agreement with the data reported in similar human models [62]. Likewise, Le Roux and collaborators [63] obtained high correlations between the drug permeability across an in vitro human iPSC-derived model and the ratio of plasma to brain permeability in patients, measured via positron emission.

The behavior of these molecules across the BBB model indicated the expected differences in permeability, suggesting the use of this platform for the evaluation of drug transport mechanisms.

### 2.7. Responsiveness of the BBB Models to Immune Factors

**Figure 9:**
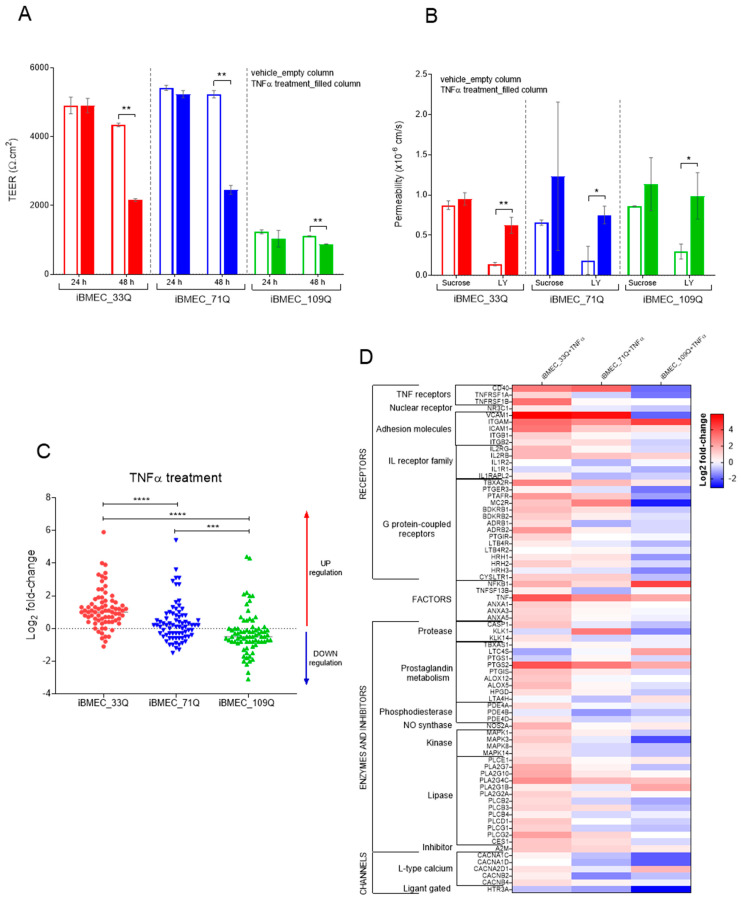
Responsiveness of the BBB Models to TNFα. (**A**) TEER measures after 24 h and 48 h of TNFα treatment (filled columns) and control cells (empty columns); *n* = 3. (**B**) Paracellular markers permeability after 48 h of TNFα treatment (filled columns) and control cells (empty columns); *n* = 3. Statistical analysis (for TEER and Permeability) by Student’s *t*-test: * *p* < 0.05 and ** *p* < 0.01. (**C**) Change in the expression of selected genes in iBMECs after TNFα treatment; *n* = 75. Statistical analysis: two-way ANOVA followed by Bonferroni post hoc test, where *** *p* < 0.001 and **** *p* < 0.0001 and (**D**) heat map of the expression by family. Values are expressed as Log2 fold change expression after treatment reported to the untreated cells for each gene normalized to GAPDH.

Neuroinflammation is the inflammation associated with neurodegenerative disease. Many clinical features of inflammation are present in HD, such as the activation of microglia, immune activation in CSF, signs of oxidative stress in post mortem brains, and alteration in the function of the peripheral immune system [64]. The BBB models expressed cytokine and chemokine receptors as well as different toll-like receptor isoforms. To evaluate their functionality in the presence of neuro-inflammatory mediators, cells were treated with TNFα and subjected to a transcription profile of a panel of inflammatory markers genes. TNFα exposure for 48 h resulted in decreased tightness as reflected by the reduction in TEER values in the iBMECs 33Q and 71Q. A much lower reduction was measured in the iBMEC_109Q cells, which were not able to create a barrier as tight as that of iBMECs 33Q and 71Q in unstimulated conditions (Figure 9A). The decrease in tightness as measured by TEER was accompanied by an increase in LY permeability (Figure 9B).

Cell stimulation with TNFα changed the expression of genes mainly associated with cellular development, cell death, and survival, such as GPCRs and tissue morphology. In the resting state, some genes were differentially expressed between healthy and HD cells, particularly *CD40* (>2 fold in both iBMEC_71Q and 109Q vs. iBMEC_33Q) and *VCAM-1* (2 fold, iBMEC_109Q vs. iBMEC_33Q), indicating a “pre-activated state” in 109Q in particular. VCAM1 plays a role in neurothophil migration into the CNS by opening the pores in the BBB and allowing the cells to enter into the brain. Endothelial cells normally express low levels of VCAM1 that can be upregulated in inflammatory conditions [65]. The high levels found in the iBMEC_109Q models may be indicative of an inflamed state. Furthermore, we observed a strong upregulation of *VCAM1* in iBMECs 33 and 71Q after treatment with TNFα (more than 40-fold) and none in iBMEC_109Q. TNFα treatment also upregulated *ICAM-1* expression in all iBMECs (9-fold in 33Q, and about 2-fold in 71 and 109Q); these data agree with the results obtained by other authors for healthy iBMECs [66,67]

The iBMEC_109Q cells responded to TNF by an overall downregulation (Figure 9C) by opposition to the healthy cells (overall upregulation), with the iBMEC_71Q having an intermediate profile. This would confirm the idea of an activated state partially incompetent to new inflammatory stimuli. However, the “pre-activated” iBMEC_109Q state was not really superimposable to the healthily activated state (Figure 9D), indicating that the TNFα pathway is probably not the only pathway responsible for the peculiar HD-iBMEC’s status.

These results suggested that this model can be useful for inflammatory modelling to study the function of pathological mediators of neuroinflammatory disorders, their role in the disease, and how to potentially enhance host-protective mechanisms.

## 3. Discussion

Most of the current knowledge about the barrier function of the BBB has been acquired from in vivo experiments, but this evaluation has proven to be challenging [17]. PET imaging with ^11^C-radioisotopes is a technique that evaluates in vivo BBB transporter function in humans, while knockout and transgenic animal models can be employed to understand the molecular mechanism of the transporters. Nonetheless, variation in selectivity and sensitivity of probes used, as well as species differences, has stimulated the development of alternative models. In addition, mechanistic studies on the barrier function and interactions with drugs at molecular and cellular levels are difficult to perform in vivo, and it might be best suited to be investigated by using in vitro systems.

In an attempt to create diseased BBB-like models, we have used iPSC cell lines derived from HD patients and we fully characterized the paracellular and transcellular transport in comparison with a control cell line. All iPSC-derived model were able to successfully differentiate in brain endothelial cells within 12 days, with barrier properties and endothelial markers with the proper subcellular organization and function. These static transwell models showed significantly higher TEER values than several on-chip models and within the range of in vivo levels [68], suggesting that shear stress is not strictly required for the acquisition of in vivo barrier functions. They recapitulated the BBB function in terms of phenotypes and functional passive and active barriers, without applying shear stress. Functional testing with CNS compounds covering a wide range of physico-chemical properties showed that the model exhibited selective permeability of both passively diffused substances and substances that interacted with brain-specific transporters.

The use of human brain microvascular endothelial cells derived from iPSCs in our BBB models enabled the reproduction of BBB-specific endothelial characteristics, resulting in a tight barrier with permeability comparable to recent tri-culture or advanced 3D models.

Although the predictive capacity has not been fully established, the in vitro models presented here were able to mimic many key features of the in vivo BBB in healthy and disease conditions.

All cell lines were able to differentiate in brain-like endothelial cells with high TEER and low passive permeability of compounds subjected to paracellular transport, and they maintained the BBB properties for several days, allowing an investigation of the long-term effects of therapeutic agents or effectors. However, we found that HD models presented a barrier leakage, as indicated by the lower TEER and higher paracellular permeability of some small tracers. Immunofluorescence imaging of tight junction proteins such as Claudin-5 did not provide insight on their dysfunctional localization at cell–cell contacts and additional methods of evaluating structural changes such as electron microscopy may be required [69]. The cellular morphology was organized with a continuous pattern of proteins of the TJs, and the monolayer was asymmetrically polarized, as indicated by the transport of some classical substrates of ABC and SLC transporters.

However, differential gene expression trends for some proteins of tight junction in healthy and HD models were quantified. Further immunoassay analysis revealed that the HD BBBs were characterized by a dysfunction in the barrier-forming Claudin 5 and Claudin 1 tight-junction proteins. These differences in expression levels could be responsible for the overall paracellular transport characteristics of both 71Q and 109Q models.

In addition to the tight junctions, all models were characterized by the presence of both specific transport system carriers and receptors. Transport systems present in the BBB and detected in vitro included those for amino acids, peptides, hexoses, monocarboxylic acids, organic cations, nucleosides, vitamins, and various xenobiotics. Many of these transporters facilitate the transcellular passage of specific solutes that are both necessary for CNS homeostasis and are unable to be synthesized de novo within this compartment. All models showed functional activities of efflux transporters such as Pgp and BCRP, with a net basal (brain) to apical (blood) transport of substrates, inhibited by the co-application of the respective inhibitors.

Whole genome expression profiling revealed transcriptional changes that occur in the HD models.

Key SLC transporters such as glucose transporter 1, GLUT-1 (*SLC2A1*), large neutral amino acid transporter 1, LAT-1 (*SLC7A5*) and monocarboxylate transporter 1, MCT1 (*SLC16A1*) showed comparable functional activity in the healthy and in the HD models. We observed a significant downregulation of the RNA of *SLC16A4* (MCT5) and *SLC16A10* (MCT10) in the 71Q-derived model, and it was more pronounced in the 109Q-derived model. While the substrate of MCT5 is unknown [70], MCT10 is reported to be an active thyroid hormone transporter but its physiological relevance for thyroid hormone action and metabolism in different tissues as the brain remains to be elucidated [71].

In the whole transcriptome data set, 440 *SLCs* were analyzed/tagged, of which 395 genes were detected in at least one iBMECs. About 48% (190) were expressed in at least one cell line over a threshold of 10 CPM, this value being set as the cut-off for reliable expression.

Among them, the highest expression was measured for *SLC2A3* (GLUT3). In compliance with the very high energy need of the brain, the other glucose transporter *SCL2A1* (GLUT1) was also expressed at high levels. Among the 25 most expressed *SLC* genes (Table S2), we found transporters for sugars, amino acids, cations, nucleotides, and other products of the metabolism machinery. Of note, many of them are mitochondrial transporters. Many of them were dysregulated in HD, either down (<0.8 FC: *SCL7A5, SLC25A6, SLC44A2, SLC2A4RG* (transcription factor), *SCL11A2*) or up (>1.2 FC: *SLC16A1, SLC1A5, SLC7A1*). *SLC7A1*, a large neutral amino acid transporter subunit, has been linked to autism disorder [34] and is already picked up as dysregulated in HD endothelial cell lines [12].

For the SLCO family, *SLCO2A1* showed higher expression. This member is important because *SLCO* seems to be expressed on both sides of the BBB, and this is a critical consideration for drug delivery to the brain mediated by this family [72].

In this situation, it might be expected that a drug can enter the endothelial cell via the luminal transporter and then exit via abluminal transporters with a net effect similar to facilitated diffusion. Therefore, the rate of transendothelial transport will depend upon the relative expression of the isoform at the luminal and abluminal plasma membrane of the BBB.

Because other types of transport mechanisms are hypothesized to be altered in disease such as AD [73], we used the HD disease models to understand changes to receptor-mediated transport that may occur. Transferrin is among the most abundant protein in *human* blood and is transported across the BBB by receptor-mediated transcytosis [74]. Active blood-to-brain transport of transferrin was recapitulated in all models, but it was significantly higher in the HD models. The contribution of the leakiness of the TJs and mechanisms of the intracellular sorting to the overall high rate of receptor transcytosis of transferrin needs to be further evaluated.

All models expressed specialized enzymes for the degradation of multiple substrates including cytochromes P450 (CYPs450), monooxygenases (phase I enzymes), monoamine oxidase, glutathione-S-transferases (*GST*), methyltransferases, UDP-glucuronosyltransferases (UGT), methyltransferases (phase II enzymes) as the catechol-O-methyltransferase (COMT), and gamma glutamyl transpeptidase (γ-GT), which has been described as highly active in BBB [37]. Alkaline phosphatases, reported to be a salient markers of a maintained BBB phenotype in cultured BMECs [75], were also present.

The BBB models displayed sensitivity to immune signals, as demonstrated by the effect of the proinflammatory cytokine TNFα, in agreement with the expression of receptors that enable them to respond to systemic inflammation. Barrier properties decreased after exposure to TNFα, as already observed by Vatine et al. [23]. In the steady-state conditions, the healthy BBB model expressed low levels of molecules involved in the transmigration of leukocytes and T-cells into the brain, such as *VCAM-1* and *CD40*, compared with the HD models. However, it has not been evaluated if the sustained inflammatory conditions for the HD BBB systems could enhance the cellular traffic and infiltration of innate immune cells.

Recently, it has been reported that iPSC-derived brain endothelial cells may display a mixed endothelial epithelial phenotype [76]. Despite this mixed endothelial–epithelial transcriptional profile, these cells expressed major BBB properties, and they constituted a valuable tool of permeability assessment in drug discovery.

The BBB is a complex multicomponent structure that is likely to have several critical requirements for in vitro culture to correctly model its functions. The optimization of iPSC-derived BBB models needs to be exploited to further produce systems with more similar signature to brain endothelial in vivo. Using human brain-specific cells provides an opportunity to evaluate different transport routes that could not have been identified with the current standard tools in vitro or in vivo. The earlier prediction of brain exposure by a combination of mechanisms would generate better translatability to in vivo conditions.

However, many of the results from experiments in this work and in other BBB in vitro platforms warrant further experimental verification and confirmation via in vivo studies, although such validation is challenging due to the biological complexity of the system.

Blood–brain barrier models such as those developed in this work could be considered complementary tools designed to support basic and translational studies in CNS drug discovery and cerebrovascular diseases. Our BBB platforms may provide a reliable tool for a better understanding of drug distribution and efficacy at the BBB in both physiological and pathological conditions. Considering the ability to recapitulate certain human functions or pathological mechanisms, the HD models can be a useful standardized tool to study the HD biology and a platform for moderate-throughput drug screening of drugs in the context of the disease.

## 4. Materials and Methods

### 4.1. Reagents

Fetal Bovine Serum (FBS), Penicillin/streptomycin, glutamine, Dextran, Texas Red™ (3000, 10,000, 40,000 and 70,000 MW), B-27™ Supplement (50X), HEPES and Hoechst 33342 were from Invitrogen (Thermo Fisher, Monza, Italy). [^14^C]sucrose, [^14^C]mannitol, [^3^H]methyl-glucose, [^3^H]citalopram, [^14^C]leucine, [^14^C]phenytoin, [^14^C]phenylalanine, [^14^C]caffeine, [^3^H]daunomycin, [^3^H]testosterone, [^3^H] [D-Ala2]-Deltorphin II, [^3^H]propranolol, [^3^H]verapamil, [^3^H]prazosin and Microscint 20 Scintillation liquid were purchased from Perkin Elmer, Milan, Italy. [^3^H]vinblastine, [^3^H]L-DOPA, [^3^H]bupropion, [^14^C]taxol, [^3^H]gabapentin, [^3^H]indomethacin, [^3^H]raclopride, and [^3^H]flumazenil are from BIOTREND Chemikalien GmbH (Köln, Germany). Dimethyl sulfoxide (DMSO), lucifer yellow (LY), kynurenic acid, (GF120918), KO143, JPH203, phloretin, and triton X100 were purchased from Sigma-Aldrich, Milan, Italy. Aqueous solutions of 8% paraformaldehyde were obtained from Electron Microscopy Sciences (Hatfield, PA, USA).

Tissue-culture-treated multi-well plates and transwell filter inserts (1.12 cm^2^ growth area, 0.4 µm pore size; transparent polyester) and Corning 96-well plates polystyrene were obtained from Corning (Sigma-Aldrich, Milan, Italy). Optiplate-96 and white Opaque 96-well microplate were from Perkin Elmer, Milan, Italy.

### 4.2. Cell Culture

Cell Culture. HUV-EC-C (HUVEC) cells were obtained from ATCC, Manassas, Virginia, USA (ATCC^®^ CRL1730™) and maintained in F12K Medium (ATCC 302004) supplemented with 10% heat-inactivated FBS, 100 U/mL penicillin, 0.1 mg/mL heparin (Sigma, Milan, Italy, cat. #H3393), and 0.3 mg/mL Endothelial Cell Growth Supplement—ECGS (Thermo Fisher, Monza, Italy, cat. #CB40006)—at 37 °C in a 5% CO_2_ atmosphere. Culturing procedures were performed according to vendor’s product sheet.

### 4.3. iPSCs Culture and Characterization

The iPSC lines used in this study were obtained from RUCDR Infinite Biologics (iPS Academia Japan, Inc., Kyoto), (Cell line ID: NN0004300_33Q, NN0000032_71Q, and NN0000037_109Q) from females and with heterozygous and altered CAG lengths (33, 71 or 109). Each clone was expanded and cryopreserved in the Working Cell Bank using standardized internal procedures. All cell lines were maintained at 37 °C, 5% CO_2_, and cultured using feeder free conditions on Matrigel (Corning from Sigma-Aldrich, Milan, Italy)-coated surfaces plates in mTeSR1 medium (STEMCELL Technologies, Cambridge, UK)) and gently dissociated with 1 U/mL dispase (BD Biosciences, Milan, Italy), at 70% confluency every 4–5 days. The working cells banks were routinely subjected to analysis of genomic stability by using karyotyping (ISENET Biobanking service unit in Milan, Italy) and to the expression of markers associated with pluripotency (Oct-4) by flow cytometry.

### 4.4. Generation of iBMECs

The differentiation was performed in about 12 days (Figure S2) as previously described [16] with some modifications, such as serum free conditions: using B-27 as a serum-free supplement instead of PDS (platelet-poor plasma-derived bovine serum) for human endothelial cell medium and instead of KORS (KnockOut Serum Replacement) for the unconditioned medium.

### 4.5. Uptake of LDL and Immunofluorescence of LDLR

Cells differentiated at day10 (D10) were used for transport studies in the monolayer with the LDL Uptake Assay Kit (Abcam, Milan, Italy, ab133127). Culture medium was aspirated and replaced with LDL-DyLight 550 working solution. Cells were then incubated for 4 h at 37 °C in a 5% CO_2_ atmosphere, followed by three washes with sterile PBS, and then visualized by INCELL-6000 (GE Healthcare, Rome, Italy) with excitation and emission wavelengths of 540 and 570 nm, respectively.

After visualization, cells were fixed with a cell-based fixative solution for 10 min. Cells were then washed with tris buffered saline plus 0.1% Triton X-100 for 5 min, followed by 30 min blocking with Cell-Based Assay Blocking Solution. Cells were then stained with *rabbit* anti-LDL receptor primary antibody and DyLight 488–conjugated secondary antibody. Images were taken with a fluorescent microscope and with excitation and emission wavelengths of 485 and 535 nm, respectively.

### 4.6. HTT Quantification by Singulex Assay

Cell pellets were lysed in TBS 0.4% Triton X-100 supplemented with protease (Roche cat. #11697498001) and phosphatase inhibitor cocktail (Roche, Indianapolis, IN, USA, cat. #04986837001) in a ratio of 250 µL of lysis buffer per 3 × 10^6^ cells. Cell lysates have been sonicated, clarified through centrifugation, and quantified by BCA protein assay kit (Thermo Fisher, Monza, Italy, cat. #A53225). The Singulex assay was performed as follows: 50 µL/well of dilution buffer (6% BSA, 0.8% Triton X-100, 750 mM NaCl) supplemented with protease inhibitor cocktail was added to a 96-well plate (Axygen, Sigma Aldrich, Milan, Italy, cat. #P-96-450V-C). Cell lysates were diluted in artificial cerebral spinal fluid (ACSF: 0.3 M NaCl; 6 mM KCl; 2.8 mM CaCl_2_-2H_2_O; 1.6 mM MgCl_2_-6H_2_O; 1.6 mM Na_2_HPO_4_-7H_2_O; 0.4 mM NaH_2_PO_4_-H_2_O) supplemented with 1% Tween-20 and protease inhibitor cocktail, and for each sample, a serial dilution curve (6 dilution points 1:3 plus blank, technical duplicates) was performed in ACSF starting from 0.05 µg/µL in a final volume of 150 µL/well. For the capturing step, 100 µL/well of the anti-HTT *N*-terminal domain antibody (2B7, obtained from the CHDI Foundation, Los Angeles, CA, USA) coupled with magnetic particles diluted in Erenna Assay buffer (Merck, Milan, Italy, cat. #02-0474-00) at a final concentration of 0.025 mg/mL was added to the assay plate and incubated for 1 h at room temperature under orbital shaking. The beads were then washed with Erenna System buffer (Merck, Milan Italy, cat. #02-0111-00) and resuspended using 20 µL/well of the specific detection antibody labelled with Alexa-647 fluorophore diluted in Erenna Assay buffer at a final concentration of 0.5 ng/µL (anti-HTT polyQ antibody, MW1 obtained from the CHDI Foundation, Los Angeles, CA, USA; anti-HTT antibody, D7F7 commercially available from Cell Signalling Technologies, Danvers, MA, USA, cat. #5656). The plate was incubated for 1 h at room temperature under shaking. After washing, the beads were resuspended and transferred into a new 96-well plate; 10 µL/well of Erenna buffer B (Merck, Milan, Italy, cat. #02-0297-00) was added to the beads for elution and incubated for 5 min at room temperature under orbital shaking. The eluted complex was magnetically separated from the beads and transferred into a 384-well plate (Sigma, Milan, Italy, cat. #264573) where it was neutralized with 10 µL/well of Erenna buffer D (Merck, Milan, Italy, cat. #02-0368-00). Finally, the 384-well plate was heat-sealed and analyzed with the Erenna Immunoassay System. The 2B7 antibody was conjugated to magnetic particles following the manufacturer’s protocol (Merck, Milan, Italy, cat. #03-0077-02), while the Alexa-647 labeling for MW1 and D7F7 antibodies was performed using the Alexa Fluor-647 Monoclonal Antibody Labelling Kit from Thermo Fisher, Monza, Italy (cat. #A20186) according to the manufacturer’s instructions.

### 4.7. Flow Cytometry

Singularized cells were fixed and permeabilized with a commercial fixation buffer: Transcription Factor Buffer Set (BD Pharmingen™, cat. #562574 BD from S.I.A.L. Srl, Rome, Italy) according to the manufacturer’s protocol. Cells were incubated with human Oct-4A, Von Willebrand Factor antibody or isotype control antibodies (see Table S5). The cell suspension was analyzed on a BD FACS Canto II Flow cytometer. Isotype-match cells were used as the control. Data were analyzed with FCS Express software (version 5.0.85).

### 4.8. Immunocytochemistry

Cells in transwell inserts (polyester membrane Transwell-Clear) were washed with phosphate buffered saline (PBS) and fixed in 4% paraformaldehyde for 20 min at room temperature (except for PECAM1 for which cold methanol (VWR Chemicals, Milan, Italy) fixation was used). Cells were permeabilized by washing with PBS/0.1% Triton X-100 and blocked in PBS containing 1% bovine serum albumin (blocking buffer) for 2 h at room temperature. Primary antibodies diluted as reported in Table S5 were added in blocking buffer for 2 h at room temperature. Cells were then incubated in a blocking buffer with the secondary antibody, diluted as recommended by the manufacturer, and the nuclear stain Hoechst 33342 at 2 µM (Thermo Fischer, Monza, Italy) was applied for one hour at room temperature.

### 4.9. Western Blot

Cells were lysed in a RIPA buffer (300 mM NaCl, 10 mM TrisHCl pH 8.0, 10 mM KCl 1 mM EDTA, 1% Nonidet-P40, 1% Na Deoxycholate, 0.1% SDS, 1 mM PMSF) and protease inhibitors cocktail (11697498001, Roche, Indianapolis, IN, USA), sonicated on ice with a Branson 450 sonicator through two cycles of ten 2 s pulses (1 min pause between cycles), and then cleared by centrifugation at 13,000× *g* for 10 min. Equal amounts of protein were separated by 4–12% gradient gels (Life Technologies, Great Island, NY, USA) and transferred to nitrocellulose membranes (10401396, Whatman, Maidstone, UK). Membranes were blocked with 5% milk in TBST (150 mM NaCl, 10 mM Tris HCl pH 7.5, 0.1% Tween 20) for one hour at room temperature. Immunostaining was accomplished by overnight incubation with primary antibodies (Table S5) followed by one hour of incubation with dye-conjugated secondary antibodies according to manufacturer’s instructions. Protein detection was achieved by using an Infrared Odyssey system (LiCor, Lincoln, NE, USA). Densitometric analysis of Western blots was performed by using ImageJ software. Actin was used as normalization probe.

### 4.10. mRNA Extraction and Quantitative Real-Time PCR

To evaluate the self-renewal capacity and the pluripotency as well as the trilineage differentiation potential, mRNA was subjected to the hPSC scorecard assay (Cat. A15872, Thermo Fisher, Monza, Italy) after RNA extraction from iPSC colonies with RNeasy mini kit (Cat. 74106, Qiagen, Hilden, Germany). cDNA was obtained with High-capacity cDNA reverse transcription kit (Cat. 4374966, Thermo Fisher, Monza, Italy). The expression of inflammatory genes after TNFα treatment was evaluated using the TaqMan™ Array *Human* Inflammation Panel (Cat. 4378722, Applied Biosystem, Foster City, CA, USA). qRT-PCR was performed in QuantStudio 12 K Flex (Thermo Fisher, Monza, Italy). The analysis of the hPSC score was conducted with the hPSC Score Card Panel software.

To mimic a neuroinflammatory condition, cells in transwells were treated with 50 ng/mL of TNFα for 48 h before RNA extraction.

### 4.11. Transcriptome Analysis

iBMECs were lyzed at day1 co-culture in supplied RLT buffer containing 1% β-mercaptoethanol. Total RNA was extracted with the RNeasy mini kit (as above) following manufacturer’s protocol. Samples were purified from genomic DNA by an additional step of DNAse I digestion (DNase Max KIT Qiagen, Hilden, Germany). RNAseq was performed by the provider (Genomnia srl, Bresso, Italy), using Ion AmpliSeq technology, allowing the evaluation of over 20,000 genes. The output value for the detected expression is the normalized counts per million (CPM) value. The differentially expressed mRNAs were collected by R package edgeR (version 3.20.9) [77] with the differential expression |log2 (fold change)| > 1, *p*-value < 0.05 and FDR ≤ 0.05. Data were deposited at ENA’s Sequence read Archive with Access number PRJEB49487.

### 4.12. TEER Measurement and Transport Assay

The measurement of TEER, transport studies, and the derivation of permeability coefficients were carried out as reported [78]. In each filter, a paracellular marker (radiolabelled sucrose or LY) was added as internal control of the tightness of the monolayer. The amounts of radiotracer and fluorescent tracers were determined by liquid scintillation (Top Count-NXT, Microplate Scintillation and Luminescence counter from Perkin Elmer, Milan, Italy) and fluorescence spectrophotometry (SAFIRE TECAN, Microplate Fluorescence reader, Männedorf, Switzerland), respectively. In efflux and influx transport assays, before the addition of the compounds, filters were pre-incubated for 15 min at 37 °C with or without inhibitors and/or substrates: 2 µM elacridar (PGP inhibition), 2 µM of KO143 (BCRP inhibition), 10 µM JPH203 (LAT-1 inhibition), and 200 µM of phloretin (GLUT inhibition). Each test compound was assayed in triplicates. Transferrin from *Human* Serum, Alexa Fluor™ 594 Conjugate (T13343 Invitrogen™ from Thermo Fisher, Monza, Italy) was incubated (2.5 µM) in HBSS-20 mM Hepes pH 7.4 containing 1% BSA for 2 h, at 37 °C, and 5% CO_2_ and on ice at 4 °C. At the end of the incubation, aliquots from both compartments were collected and the fluorescent tracer was quantified.

### 4.13. Statistical Analysis

Data were presented as mean ± standard deviation or mean ± standard error. Statistical analyses (Prism 8.4.3, GraphPad, San Diego, CA, USA) were performed using an unpaired, two-tailed Student’s *t*-test or analysis of variance (ANOVA). The value of *p* <  0.05 was taken as the criterion for statistically significant differences.

## Figures and Tables

**Table 1:** Permeability of commercially available compounds with different transport mechanisms.

Compound	BBB Transport Mechanism	Permeability (×10^−6^ cm/s)		
		*iBMEC_33Q*	*iBMEC_71Q*	*iBMEC_109Q*
arginine	active influx (y^+^ L)	9.0 ± 0.8	12.2 ± 1.0	9.2 ± 0.5
atenolol	passive diffusion	0.66 ± 0.02	0.75 ± 0.05	1.59 ± 0.16 *
bupropion ^a^	multiple mechanisms	20.6 ± 0.7	26.4 ± 1.8	25.2 ± 1.6
caffeine ^a^	passive diffusion/active influx	26.5 ± 0.3	26.6 ± 1.4	24.5 ± 1.4
citalopram	multiple mechanisms	50.0 ± 4.2	59.6 ± 1.3	74.2 ± 1.6 *
daunomycin	passive diffusion/active efflux (Pgp)	3.0 ± 0.1	2.6 ± 0.1	2.8 ± 0.2
[D-Ala2]deltorphin-II	multiple mechanisms	0.77 ± 0.08	0.92 ± 0.01	1.01 ± 0.02
flumazenil ^a^	passive diffusion/active efflux (Pgp)	23.4 ± 1.2	20.7 ± 0.5	25.7 ± 1.3
glucose ^a^	active influx (GLUT-1)	15.0 ± 0.9	15.3 ± 1.6	16.1 ± 0.7
glutamate	active efflux (x^−^)	4.1 ± 0.2	3.3 ± 0.1	7.2 ± 0.7 *
indomethacin	multiple mechanisms	18.1 ± 0.8	29.3 ± 7.2	34.0 ± 4.9
lactate ^a^	active influx (MCT1)	53.8 ± 6.7	53.2 ± 3.6	52.6 ± 5.7
L-DOPA	active influx (LAT-1)	25.1 ± 4.1	18.0 ± 2.5	18.8 ± 1.1
leucine ^a^	active influx (LAT-1)	15.7 ± 1.6	11.3 ± 0.7	19.5 ± 2.5
phenylalanine ^a^	active influx (LAT-1)	25.8 ± 1.5	21.4 ± 1.4	30.4 ± 3.0
phenytoin ^a^	passive diffusion/active efflux (MRP)	18.6 ± 0.2	20.9 ± 1.0	27.7 ± 1.5 *
prazosin ^a^	passive diffusion/active efflux (BCRP)	3.8 ± 0.5	3.9 ± 0.6	5.2 ± 0.4 *
propranolol ^a^	passive diffusion	22.0 ± 1.4	23.4 ± 1.7	17.1 ± 0.9
raclopride ^a^	passive diffusion	31.1 ± 2.6	31.6 ± 1.8	21.4 ± 0.4
taxol ^a^	Passive diffusion/active efflux (Pgp)	5.0 ± 0.2	3.5 ± 0.3	6.7 ± 0.4
testosterone	Passive diffusion	80.1 ± 17.8	72.8 ± 5.1	101.2 ± 13.9
Verapamil ^a^	Passive diffusion/active efflux (Pgp)	14.0 ± 0.9	16.9 ± 0.3	16.7 ± 1.0
vinblastine	Passive diffusion/active efflux (Pgp)	1.2 ± 0.1	1.5 ± 0.2	1.6 ± 0.1

Summary of the permeability values in the in vitro iBMECs models reported as mean ± SE with *n* > 6 from at least two separate experiments. ^a^ The value was calculated from P_total_, since P_endothelial_ exceeded P_filter_. The statistical analysis (Student’s *t*-test) for the difference of permeability for the compound alone in iBMEC_109Q vs. iBMEC_33Q is indicated by asterisks: * *p* < 0.05.

**Table 2:** Polarized transport in iBMECs.

Compound	iBMECs	Inhibitor	Permeability (×10^−6^ cm/s)		Unpaired *t*-Test	Efflux Ratio
			A-B	B-A	*p* Value (BA vs. AB)	
daunomycin	33Q	-	3.0 ± 0.1	3.9 ± 0.2	*<0.01*	**1.3**
	71Q	-	2.6 ± 0.1	3.5 ± 0.3	*<0.05*	**1.3**
	109Q	-	2.8 ± 0.2	4.3 ± 0.2	*<0.001*	**1.5**
taxol	33Q	-	5.0 ± 0.2	6.9 ± 0.4	*<0.05*	**1.4**
	71Q	-	3.5 ± 0.3	6.3 ± 0.2	*<0.01*	**1.8**
	109Q	-	6.7 ± 0.4	8.9 ± 0.4	<0.05	**1.3**
verapamil	33Q	-	14.0 ± 0.9	20.3 ± 0.8	*<0.001*	**1.5**
	71Q	-	16.9 ± 0.3	19.2 ± 0.6	*ns*	**1.1**
	109Q	-	16.7 ± 1.0	16.3 ± 1.1	*ns*	**1.0**
vinblastine	33Q	-	1.2 ± 0.1	2.8 ± 0.2	*<0.01*	**2.3**
		+2 µM elacridar	1.7 ± 0.1 *	2.5 ± 0.2	*<0.05*	**1.5**
	71Q	-	1.5 ± 0.2	2.7 ± 0.3	*<0.05*	**1.8**
		+2 µM elacridar	1.9 ± 0.1	2.0 ± 0.3	*ns*	**1.0**
	109Q	-	1.6 ± 0.1	3.1 ± 0.7	*ns*	**1.9**
		+2 µM elacridar	2.9 ± 0.6	2.9 ± 0.4	*ns*	**1.0**
prazosin	33Q	-	3.8 ± 0.5	17.1 ± 1.0	*<0.001*	**4.5**
		+2 µM KO143	14.5 ± 1.3 **	15.2 ± 0.8	*ns*	**1.0**
	71Q	-	3.9 ± 0.6	16.2 ± 1.6	*<0.001*	**4.2**
		+2 µM KO143	12.4 ± 0.9 **	17.1 ± 0.8	*<0.05*	**1.3**
	109Q	-	5.2 ± 0.4	16.7 ± 1.2	*<0.001*	**3.2**
		+2 µM KO143	7.6 ± 0.5 *	11.9 ± 0.5	*<0.01*	**1.6**
glutamate	33Q	-	4.1 ± 0.2	7.5 ± 0.5	*<0.05*	**1.8**
	71Q	-	3.3 ± 0.1	6.8 ± 0.6	*<0.05*	**2.0**
	109Q	-	7.2 ± 0.7	9.0 ± 0.5	*ns*	**1.3**
leucine	33Q	-	15.7 ± 1.7	19.4 ± 1.0	*ns*	
		+10 µM JPH203	1.8 ± 0.3 ***	2.2 ± 0.4	*ns*	
	71Q	-	11.3 ± 0.7	17.3 ± 1.7	*ns*	
		+10 µM JPH203	1.8 ± 0.1 ***	2.2 ± 0.1	*ns*	
	109Q	-	19.5 ± 2.5	25.3 ± 1.7	*ns*	
		+10 µM JPH203	3.0 ± 0.3 ***	5.7 ± 0.9	*ns*	
glucose	33Q	- +200 µM phloretin	16.7 ± 0.9 6.3 ± 0.7 ***	12.9 ± 2.5 5.2 ± 0.9	*ns* *ns*	
	71Q	- +200 µM phloretin	18.6 ± 0.7 7.2 ± 1.5 **	17.9 ± 4.3 6.2 ± 1.0	*ns* *ns*	
	109Q	- +200 µM phloretin	14.1 ± 1.6 6.5 ± 1.1 **	12.5 ± 0.9 5.8 ± 0.6	*ns* *ns*	
lucifer yellow	33Q	-	0.23 ± 0.02	0.21 ± 0.04	*ns*	**0.9**
	71Q	-	0.37 ± 0.03	0.34 ± 0.06	*ns*	**0.9**
	109Q	-	0.70 ± 0.03	0.70 ± 0.1	*ns*	**1.0**

Permeability of transported substrates across iBMECs in presence of different inhibitors. Coefficients in the A-B and in the B-A directions are reported as mean ± SD of at least 3 biological replicates along with respective efflux ratio. Statistical difference between both sides is reported as *p*-value; ns = no significant. The statistical analysis (Student’s *t*-test) for the difference of permeability for the compound alone or in the presence of an inhibitor is indicated by asterisks: *** *p* < 0.001, ** *p* < 0.01 and * *p* < 0.05.

## Data Availability

Not applicable.

## Supplementary Materials

The following supporting information can be downloaded at: https://www.mdpi.com/article/10.3390/ijms23147813/s1. References [79,80,81,82,83,84,85,86,87,88,89,90,91,92,93,94,95,96,97,98,99,100,101,102,103,104,105,106,107,108,109,110,111,112,113,114,115,116,117,118,119,120,121,122,123,124,125,126,127,128,129,130,131,132,133,134,135,136,137,138,139,140,141,142,143,144,145,146,147,148,149,150,151,152,153,154,155,156,157,158,159,160,161,162,163,164,165,166] are cited in Supplementary Materials.

## Abbreviations

iPSCs	induced pluripotent stem cells
iBMEC	(iPSC)-derived brain-like microvascular endothelial cells
