# Supplementary material for: Modelling the Human Blood–Brain Barrier in Huntington Disease
Source: Int J Mol Sci. 2022 Jul 15;23(14):7813. doi: 10.3390/ijms23147813 (PMC9321930; doi:10.3390/ijms23147813)
Supplement: Supplementary file 1 [file ijms-23-07813-s001.zip › ijms-1764856-supplementary.pdf]

Modelling the human blood brain barrier in Huntington Disease.

by

Domenico Vignone[1], Odalys Gonzalez Paz[1], Ivan Fini[1], Antonella Cellucci[1], Giulio Auciello[1] Maria Rosaria Battista[1], Isabelle Gloaguen[1], Silvia Fortuni[1], Cristina Cariulo[1], Vinod Khetarpal[2], Celia Dominguez[2], Ignacio Muñoz-Sanjuán[2] and Annalise Di Marco[1]*

[1]IRBM SpA, via Pontina km 30,6; 00071 Pomezia (Rome), Italy
[2]CHDI Management/CHDI Foundation, 6080 Center Drive, Los Angeles, CA 90045, USA

* To whom correspondence should be addressed: a.dimarco@irbm.com;

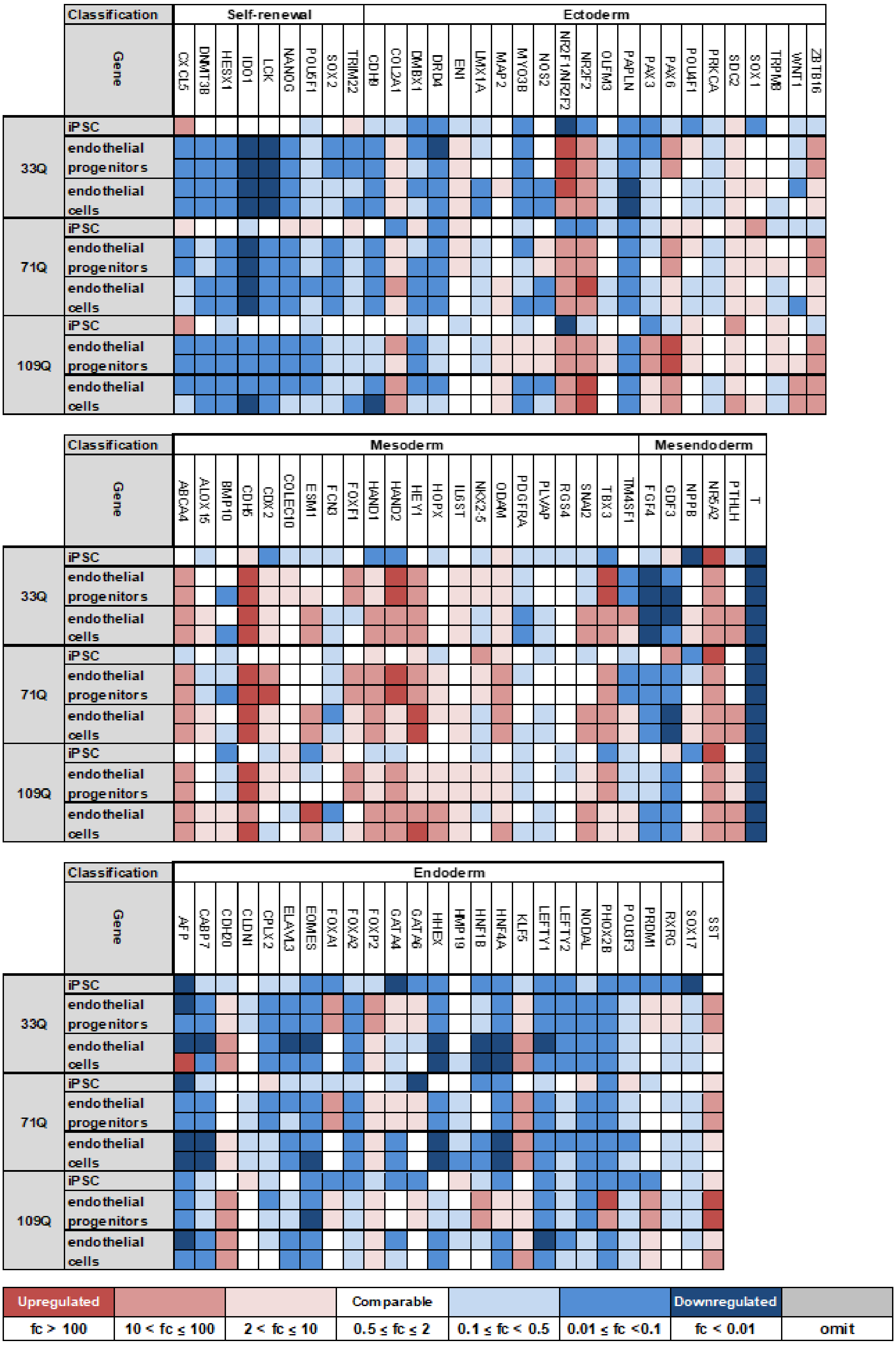

Figure S1. Characterization of human iPSCs. The pluripotency of the clones as well as their self-renewal ability were confirmed by pluripotency score card analysis. Colours indicate the fold change in expression relative to the undifferentiated reference set for each gene.

-

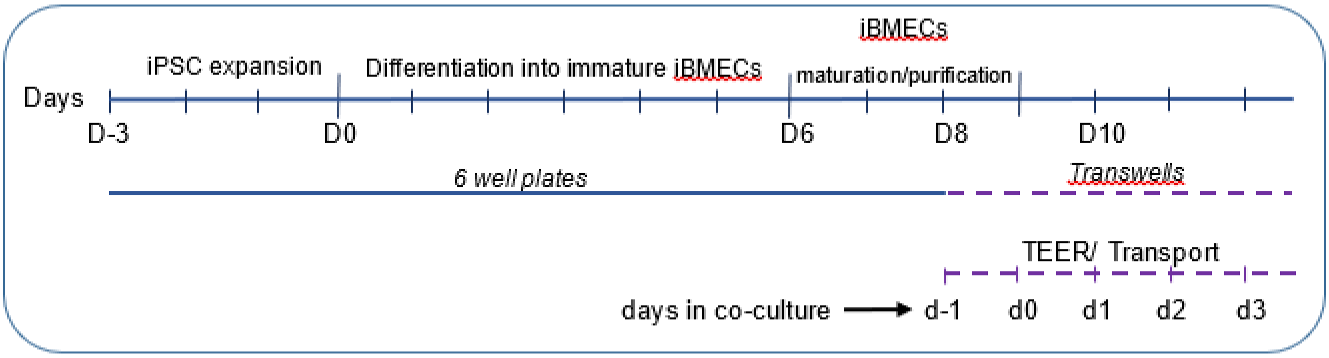

Figure S2: Generation and selection of iBMECs. Scheme illustrating the timeline and the cell culture steps.

Table S1: Differentially expressed genes between HD-iBMECs and healthy iBMEC. More than 3 fold up-regulated and down-regulated genes (statistical difference using Student's t-test set at $p<0.05$).

Gene (in use alias)	iBMEC_71 vs 33		iBMEC_109 vs 33		HD	Other neurological disorders
	FC	Log$_2$ FC	FC	Log$_2$ FC		
HLA-C	1548.8	10.6	1477.0	10.5	-	-
UNC5D	366.0	8.5	1232.1	10.3	[79]	Psychiatric illness [80]
COL22A1	763.4	9.6	678.4	9.4	[81,82]	Behavioral psychopathologies[83][a]; fragile X syndrome [84][a]
CTSF	22.4	4.5	364.3	8.5	-	adult-onset neuronal ceroid lipofuscinosis [85]
SP8	18.5	4.2	239.6	7.9	-	-
CDH6	7.0	2.8	239.4	7.9	-	AD [86]
C5orf38	167.7	7.4	142.9	7.2	-	-
LOC100128252 * (ZNF667-AS1)	>39.4	>5.3	>5	>2.3	-	Frontotemporal dementia diseases [87]; cerebral ischemia [88][a]
KCNQ2	15.3	3.9	28.5	4.8	[89,90]	Epilepsy [91]; epileptic encephalopathy [92]; Linked to CALM3 in neurological disorders [93][a]
C10orf57 (TMEM254)	21.5	4.4	24.0	4.6	-	Linked to STX1A and SYT1 in neurological disorders [93][a]
HIST1H3C	150.2	7.2	23.7	4.6	[94]	-
ZBTB16	3.5	1.8	20.7	4.4	[95]	-
IRX2*	>19.6	>4.3	>15.9	>4.0	-	PD [96]
HLA-A	5.8	2.5	17.9	4.2	[97]	Neurologicals disorders [98]
POU5F1	5.5	2.4	17.4	4.1	[99]	-
GABRB3	6.2	2.6	17.3	4.1	-	Epileptic encephalopathy [100]; neurodevelopment [101]; Febrile seizure [102]
NEFL	3.1	1.6	15.9	4.0	[103-106]	Charcot-Marie-Tooth disease [107]; PD [108]
CDH4*	>6.3	2.7	>12.4	>3.6	-	-
GAP43	6.0	2.6	11.1	3.5	-	AD and PD [109]
LIX1	4.7	2.2	10.9	3.4	-	-
ARSE	5.3	2.4	10.1	3.3	-	-
PMP22	3.9	2.0	7.7	2.9	-	Charcot-Marie-Tooth disease [110]
PCDHA11	3.8	1.9	6.9	2.8	[111]	Protocadherins in neural circuit formation [112][a]; psychosis [113][a]
DPPA4	3.3	1.7	6.9	2.8	-	-
THY1	3.7	1.9	6.8	2.8	-	-
PAX6	3.9	2.0	6.6	2.7	[114]	Autism [115][a]; neurodegeneration [116][a]

**RGL1**	**3.4**	**1.8**	**6.0**	**2.6**	-	-
**DPYSL5**	**3.2**	**1.7**	**5.6**	**2.5**	**[79,117]**	Age-related [118][a]; X-linked brain disease [119]
**SOBP**	**4.6**	**2.2**	**5.5**	**2.5**	-	Trisomy 18 [120][c]
**EFNA2**	**3.9**	**2.0**	**5.4**	**2.4**	-	PD [121]
**COL9A3**	**5.8**	**2.5**	**4.3**	**2.1**	**[122]**	Ring chromosome 20 [123]; Behavioral psychopathologies [83]
**PCDHB5**	**23.1**	**4.5**	**4.2**	**2.1**	[124[a]]	-
**KCTD12**	**3.7**	**1.9**	**3.8**	**1.9**	-	Bipolar I disorder [125]; schizophrenia [126]; depression [127]
**CENPM**	**4.1**	**2.0**	**3.7**	**1.9**	-	-
**KIAA0922 (TMEM131L)**	**3.5**	**1.8**	**3.6**	**1.9**	-	-
**SEPT3 (SEP-TIN3)**	**3.8**	**1.9**	**3.5**	**1.8**	-	AD [128]
**EEF1A2**	**3.1**	**1.6**	**3.4**	**1.8**	**[129]** *(KO induces HTT aggregation)*	Degenerative epileptic-dyskinetic encephalopathy [130]; epilectic autistic patient [131]
**CDC45**	**3.1**	**1.6**	**3.2**	**1.7**	-	Schizophrenia [132]
**ADAMTS5**	**0.3**	**-1.8**	**0.3**	**-1.8**	**[133]**	AD [134,135]; Cerebral cavernous malformation [136]
**MXRA5**	**0.3**	**-1.6**	**0.3**	**-1.9**	-	Heart failure [137]
**KCNJ2-AS1**	**0.3**	**-1.8**	**0.3**	**-1.9**	[138][a]	-
**NAALADL2**	**0.3**	**-1.7**	**0.3**	**-1.9**	-	-
**CEBPA**	**0.3**	**-1.9**	**0.2**	**-2.2**	**[139,140]**	Migraine [141][c]
**STAC**	**0.3**	**-1.6**	**0.2**	**-2.4**	**[142]**	-
**CDH20**	**0.1**	**-2.8**	**0.2**	**-2.5**	-	Glioblastoma [143]
**WNT9B**	**0.2**	**-2.3**	**0.2**	**-2.5**	**[144,145]**	Hunter syndrome [146] https://dx.doi.org/10.3390%2Fijms18051072
**FAM20A**	**0.2**	**-2.1**	**0.2**	**-2.6**	-	-
**ST6GALNAC2**	**0.3**	**-1.9**	**0.2**	**-2.6**	**[147]**	-
**TNNT3**	**0.2**	**-2.2**	**0.2**	**-2.6**	**[148]**	-
**ESRRG**	**0.2**	**-2.4**	**0.2**	**-2.6**	**[148]**	Common neurodegenerative hub [149]; AD [150][c]
**PLAGL1 (Zac1)**	**0.2**	**-2.2**	**0.2**	**-2.7**	**[151][c]**	AD [152]
**CA10**	**0.1**	**-3.0**	**0.1**	**-2.8**	-	Glioma [153]
**SLC16A10**	**0.3**	**-1.7**	**0.1**	**-3.3**	-	-
**SNCG**	**0.2**	**-2.4**	**0.1**	**-3.6**	-	PD [139,154[b]]
**NPR3**	**0.1**	**-2.8**	**0.1**	**-4.2**	-	AD [155][a]
**MRPS21**	**0.2**	**-2.6**	**0.1**	**-4.2**	-	-
**DSCR6 (RIP-PLY3)**	**0.2**	**-2.4**	**0.0**	**-4.4**	-	-
**LOC100134868**	**0.3**	**-1.6**	**0.0**	**-5.8**	-	-

a: not a strong or direct evidence,
b: absence of correlation or discorrelation
c: opposite effect observed

Table S2: Excel file transcriptome results

Table S2_selected transcriptome results.xlsx

Table S3: The most expressed SLC transporters is reported.

Gene (protein)	iBMEC			FC (iB-MEC_109 vs 33)	p value (Student's t-test)	Role	Disease
	33Q	71Q	109Q			(IUPHAR)	(Hu. 2020)
**SLC2A3 (GLUT3)**	3473	3504	3118	0.9	0.246	Brain/neuron glucose transporter	
**SLC7A5 (LAT1)**	1210	824	817	0.7	0.132	Large neutral aa subunit	Autism secptrum disorder (glioma in rodent)
**SLC25A3 (MPCP)**	1101	776	1000	0.9	0.234	Mitochondrial phosphate carrier	(11 of the- 25 family are linked to brain disorders)
**SLC25A6 (ANT3)**	633	582	501	0.8	**0.023**	Mitochondrial adenine nucleotide translocator 3	(11 of the- 25 family are linked to brain disorders)
**SLC2A1 (GLUT1)**	588	360	491	0.8	0.168	Erythrocyte /brain glucose transporter	Glucose transport type 1 deficiency syndrome. intractable infantile seizure. complex motor disorder. intellectual impairment. hypoglycorrhachia. microcephaly. (epilepsy and metabolic dysfunction in redent)
**SLC44A2 (CTL2)**	454	374	349	0.8	**0.005**	Chloline transporter-like 2	
**SLC25A5 (ANT2)**	412	467	425	1.0	0.729	Mitochondrial adenine nucleotide translocator 2	Intellectual disability
**NPC2**	386	321	318	0.8	**0.014**	NPC intracellular cholesterol transporter 2	Niemann-Pick disease, type C
**SLC39A1 (ZIP1)**	311	273	253	0.8	0.082	Zinc transporrter-1	
**SLC38A2 (SNAT2)**	294	310	286	1.0	0.840	Amino acid transporter 2	
**SLC39A7 (RING5)**	279	202	224	0.8	0.096	Zinc transporrter-7	
**SLC3A2 (4F2hc)**	264	265	249	0.9	0.153	Heterodimerize with SLC7 family members (dibasic and neutral aa)	
**SLC25A8 (UCP2)**	258	208	123	0.5	**0.003**	mitochondrial uncoupling protein 2	
**SLC16A1 (MCT1)**	233	327	333	1.4	0.103	Transport of the product of cellular metabolism	
**SLC25A1 (CIC)**	222	197	214	1.0	0.709	Mitochondrial citrate transporter	
**SLC5A6 (SMVT)**	190	207	195	1.0	0.740	Multivitamin transporter (apical membrane enterocytes and colonocytes (biotin. pantothenic acid)	
**SLC25A39 (CIG69)**	189	149	174	0.9	0.521	Mitochondrial aa transporter subfamily	Childhood absence epilepsy
**SLC35A4 (MGC2541)**	184	179	167	0.9	0.465	For nucleotide-conjugated sugars within Golgi for glycoprotein formation	
**SLC16A10 (TAT1)**	178	56	18	0.1	**0.001**	Aromatic aa (tryptophane. phenylalanine. tyrosine. L-DOPA)	
**SLC9A3R1**	168	128	174	1.0	0.829	(SLC9A3: sodium hydrogen exchanger)	
**SLC64A1 (TMEM165)**	166	135	131	0.8	**0.032**	Golgi Ca^{2+}/H^{+} exchangers	

**SLC56A1 (SFXN1)**	148	147	140	0.9	0.663	Mitochondrial serine transporte	
**SLC25A50 (MTCH2)**	141	139	135	1.0	0.557	Mitochondrial Carrier 2	
**SLC53A1 (XPR1)**	133	136	108	0.8	0.163	Xenotropic And Polytropic Retrovirus Receptor 1	Brain calcification
**SLC2A4RG**	129	113	105	0.8	**0.020**	(SLC2A4: insulin-responsive glucose transporter. GLUT4)	
**SLC58A2 (TUSC3)**	129	121	128	1.0	0.951	Tumor Suppressor Candidate 3	
**SLC11A2 (DMT1)**	113	64	66	0.6	**0.020**	Divalent cations across endosomal membranes	Parkinson’s disease in rodent
**SLC25A23 (APC2)**	105	112	99	0.9	0.616	Mitochondrial phosphate carrier 2	
**SLC35B1 (UGTREL1)**	103	92	91	0.9	0.264	Galactose transporter	
**SLC1A5 (ASCT2)**	99	115	138	1.4	**0.012**	Neutral aa transporter (alanine. serine. cysteine) (unlike EAATs same SLC1 family. donot counter transport K+)	Schizophrenia in rodents
**SLC54A2 (MPC2)**	96	96	95	1.0	0.931	Mitochondrial Pyruvate Carrier 2	
**SLC7A1 (CAT1)**	96	99	115	1.2	**0.046**	Y+ basic aa (arginine. lysine. histidine. ornithine)	
**SLC66A5 (MPDU1)**	94	79	65	0.7	**0.011**	Mannose-P-Dolichol Utilization Defect 1	
**SLC25A4 (ANT1)**	92	102	94	1.0	0.684	Mitochondrial adenine nucleotide translocator 1	Bipolar disorder in rodent

Table S4: *In vitro-in vivo* correlation. Table showing values used for correlation shown in **Figure 9** and references for in vivo $K_{p,uu,CSF}$.

**Compound**	**Human $K_{p,uu,CSF}$**	**References**
arginine	0.28	[156]
atenolol	0.18	[157]
bupropion	1.11	[158]
caffeine	1.00	[159]
carbamazepine	0.24	[160]
citalopram	1.77	[161]
digoxin	0.31	[162]
gabapentin	0.16	[157]
glucose	0.65	[161]
IgG (MEM189)	0.002	[161]
indomethacin	0.27	[157]
KYNA	0.024	[164]
L-DOPA	0.49	[158]
leucine	0.12	[156]
methotrexate	0.02	[165]
phenylalanine	0.28	[156]
phenytoin	0.19	[166]
propranolol	0.42	[157]
verapamil	1.13	[157]

Table S5: Antibodies used in this study.

Target epitope	Species	Type	Label	RRID*	Vendor	Cat n	Dilution
Claudin-5	rabbit	polyclonal		AB_2533157	Thermo Fisher	34-1600	1:25
Claudin-1	rabbit	polyclonal		AB_2533977	Thermo Fisher	71-500	1:25
Actin	mouse	monoclonal		AB_2533147	Thermo Fisher	33-9100	1:200
ACE2	rabbit	polyclonal			Abcam	Ab15384	1:500
LDLR	rabbit	polyclonal			Abcam	Ab133127	1:1000
EGFR	rabbit	monoclonal			Abcam	Ab52894	1:1000
LRP1	rabbit	polyclonal			Origene	AP21130PU-N	1:500
HAP1	mouse	monoclonal			Origene	TA309681	1:500
TFR1	rabbit	polyclonal			Origene	TA324761	1:1000
Human Oct-4A	mouse IgG2A	monoclonal			ReD System	MAB17591	10 µl x 10^6 cells
Human Von Willebrand Factor	rabbit	polyclonal		AB_2315602	Dako/Agilent	A008202-5	1:100
Von Willebrand Factor	mouse IgG1 Kappa	monoclonal	Alexa Fluor 488		Novusbio	NBP2-34510AF488	5µL x 10^6 cells
Isotype Control APC-conjugated Antibody Clone # 20102	mouse IgG2A	monoclonal			ReD System	IC003A	10µl x 10^6 cells
Isotype Control (11711)	mouse IgG1		Alexa Fluor 488		ReD System	IC002G	5µL x 10^6 cells
ZO1 tigh junction protein	mouse	monoclonal		AB_2533147	Thermo Fisher	339100	1:100
CD31/PECAM-1	rabbit	polyclonal			Thermo Fisher	RB-10333-P1	1:25 MEOH
LDLR	rabbit	polyclonal			Invitrogen	PA5-22976	1:1000
Anti-rabbit IgG (H+L) Highly Cross-Adsorbed	goat	polyclonal	Alexa Fluor 488	AB_2576217	Thermo Fisher	A-11034	1:3000
Anti-rabbit IgG (H+L) Highly Cross-Adsorbed	goat	polyclonal	Alexa Fluor 594	AB_2534095	Thermo Fisher	A-11037	1:3000
Anti-Mouse IgG (H+L) Highly Cross-Adsorbed	goat	polyclonal	Alexa Fluor Plus 488	AB_2633275	Thermo Fisher	A32723	1:3000
Anti-Mouse IgG (H+L) Highly Cross-Adsorbed	goat	polyclonal	Alexa Fluor Plus 594	AB_2534091	Thermo Fisher	A-11032	1:3000

*: Research resource identifier.

References

79. Ring, K.L.; An, M.C.; Zhang, N.; O'Brien, R.N.; Ramos, E.M.; Gao, F.; Atwood, R.; Bailus, B.J.; Melov, S.; Mooney, S.D.; et al. Genomic Analysis Reveals Disruption of Striatal Neuronal Development and Therapeutic Targets in Human Huntington's Disease Neural Stem Cells. *Stem Cell Rep.* **2015**, *5*, 1023–1038, https://doi.org/10.1016/j.stemcr.2015.11.005.
80. Srikanth, P.; Lagomarsino, V.N.; Pearse, R.; Liao, M.; Ghosh, S.; Nehme, R.; Seyfried, N.; Eggan, K.; Young-Pearse, T.L. Convergence of independent DISC1 mutations on impaired neurite growth via decreased UNC5D expression. *Transl. Psychiatry* **2018**, *8*, 245, https://doi.org/10.1038/s41398-018-0281-9.
81. Agus, F.; Crespo, D.; Myers, R.H.; Labadorf, A. The caudate nucleus undergoes dramatic and unique transcriptional changes in human prodromal Huntington's disease brain. *BMC Med Genom.* **2019**, *12*, 1–17, https://doi.org/10.1186/s12920-019-0581-9.
82. Mehta, S.R.; Tom, C.M.; Wang, Y.; Bresee, C.; Rushton, D.; Mathkar, P.P.; Tang, J.; Mattis, V.B. Human Huntington's Disease iPSC-Derived Cortical Neurons Display Altered Transcriptomics, Morphology, and Maturation. *Cell Rep.* **2018**, *25*, 1081–1096.e6, https://doi.org/10.1016/j.celrep.2018.09.076.
83. Smagin, D.; Galyamina, A.G.; Kovalenko, I.L.; Babenko, V.; Kudryavtseva, N.N. Aberrant Expression of Collagen Gene Family in the Brain Regions of Male Mice with Behavioral Psychopathologies Induced by Chronic Agonistic Interactions. *BioMed Res. Int.* **2019**, *2019*, 1–13, https://doi.org/10.1155/2019/7276389.
84. Boland, M.J.; Nazor, K.L.; Tran, H.T.; Szücs, A.; Lynch, C.L.; Paredes, R.; Tassone, F.; Sanna, P.P.; Hagerman, R.J.; Loring, J.F. Molecular analyses of neurogenic defects in a human pluripotent stem cell model of fragile X syndrome.. *Brain* **2017**, *140*, 582–598, https://doi.org/10.1093/brain/aww357.
85. van der Zee, J.; Mariën, P.; Crols, R.; Van Mossevelde, S.; Dillen, L.; Perrone, F.; Engelborghs, S.; Verhoeven, J.; D'Aes, T.; Groote, C.C.-D.; et al. Mutated*CTSF*in adult-onset neuronal ceroid lipofuscinosis and FTD. *Neurol. Genet.* **2016**, *2*, e102, https://doi.org/10.1212/nxg.0000000000000102.
86. Ahmad, S.; Milan, M.D.C.; Hansson, O.; Demirkan, A.; Agustin, R.; Sáez, M.E.; Giagtzoglou, N.; Cabrera-Socorro, A.; Bakker, M.H.M.; Ramirez, A.; et al. CDH6 and HAGH protein levels in plasma associate with Alzheimer's disease in APOE ε4 carriers. *Sci. Rep.* **2020**, *10*, 1–13, https://doi.org/10.1038/s41598-020-65038-5.
87. Ehrlich, M.; Hallmann, A.-L.; Reinhardt, P.; Araúzo-Bravo, M.J.; Korr, S.; Röpke, A.; Psathaki, O.E.; Ehling, P.; Meuth, S.G.; Oblak, A.L.; et al. Distinct Neurodegenerative Changes in an Induced Pluripotent Stem Cell Model of Frontotemporal Dementia Linked to Mutant TAU Protein. *Stem Cell Rep.* **2015**, *5*, 83–96, https://doi.org/10.1016/j.stemcr.2015.06.001.
88. Yuan, D.; Huang, J.; Yuan, X.; Zhao, J.; Jiang, W. Zinc finger protein 667 expression is upregulated by cerebral ischemic preconditioning and protects cells from oxidative stress. *Biomed. Rep.* **2013**, *1*, 534–538, https://doi.org/10.3892/br.2013.124.

89. Cao, Y.; Bartolomé-Martín, D.; Rotem, N.; Rozas, C.; Dellal, S.S.; Chacon, M.A.; Kadriu, B.; Gulinello, M.; Khodakhah, K.; Faber, D.S. Rescue of homeostatic regulation of striatal excitability and locomotor activity in a mouse model of Huntington's disease. *Proc. Natl. Acad. Sci.* **2015**, *112*, 2239–2244, https://doi.org/10.1073/pnas.1405748112.
90. Mucha, M.; Ooi, L.; Linley, J.E.; Mordaka, P.; Dalle, C.; Robertson, B.; Gamper, N.; Wood, I.C. Transcriptional Control of KCNQ Channel Genes and the Regulation of Neuronal Excitability. *J. Neurosci.* **2010**, *30*, 13235–13245, https://doi.org/10.1523/JNEUROSCI.1981-10.2010.
91. Amadori, E.; Brolatti, N.; Scala, M.; Marchese, F.; Vari, M.S.; Ramenghi, L.A.; Madia, F.; Minetti, C.; Striano, P. Precision medicine in early-onset epilepsy: The KCNQ2 paradigm. *J. Transl. Genet. Genom.* **2020**, https://doi.org/10.20517/jtgg.2020.36.
92. Weckhuysen, S.; Ivanovic, V.; Hendrickx, R.; Van Coster, R.; Hjalgrim, H.; Møller, R.S.; Grønborg, S.; Schoonjans, A.-S.; Ceulemans, B.; Heavin, S.B.; et al. Extending the KCNQ2 encephalopathy spectrum: Clinical and neuroimaging findings in 17 patients. *Neurology* **2013**, *81*, 1697–1703, https://doi.org/10.1212/01.wnl.0000435296.72400.a1.
93. Zhang, Y.; Zhao, Y.; Song, X.; Luo, H.; Sun, J.; Han, C.; Gu, X.; Li, J.; Cai, G.; Zhu, Y.; et al. Modulation of Stem Cells as Therapeutics for Severe Mental Disorders and Cognitive Impairments. *Front. Psychiatry* **2020**, *11*, https://doi.org/10.3389/fpsyt.2020.00080.
94. Świtońska, K.; Szlachcic, W.J.; Handschuh, L.; Wojciechowski, P.; Marczak, .; Stelmaszczuk, M.; Figlerowicz, M.; Figiel, M. Identification of Altered Developmental Pathways in Human Juvenile HD iPSC With 71Q and 109Q Using Transcriptome Profiling. *Front. Cell. Neurosci.* **2019**, *12*, https://doi.org/10.3389/fncel.2018.00528.
95. Zhang, T.; Dong, K.; Liang, W.; Xu, D.; Xia, H.; Geng, J.; Najafov, A.; Liu, M.; Li, Y.; Han, X.; et al. G-protein-coupled receptors regulate autophagy by ZBTB16-mediated ubiquitination and proteasomal degradation of Atg14L. *eLife* **2015**, *4*, e06734–e06734, https://doi.org/10.7554/elife.06734.
96. Sim, H.; Lee, J.-E.; Yoo, H.M.; Cho, S.; Lee, H.; Baek, A.; Kim, J.; Seo, H.; Kweon, M.-N.; Kim, H.G.; et al. Iroquois Homeobox Protein 2 Identified as a Potential Biomarker for Parkinson's Disease. *Int. J. Mol. Sci.* **2020**, *21*, 3455, https://doi.org/10.3390/ijms21103455.
97. Madsen, H.; Nielsen, L.S.; A Sorensen, S. An association study of Huntington's disease and HLA.. *J. Med Genet.* **1982**, *19*, 452–454, https://doi.org/10.1136/jmg.19.6.452.
98. Misra, M.; Damotte, V.; Hollenbach, J.A. The immunogenetics of neurological disease. *Immunology* **2017**, *153*, 399–414, https://doi.org/10.1111/imm.12869.
99. Smith-Geater, C.; Hernandez, S.J.; Lim, R.; Adam, M.; Wu, J.; Stocksdale, J.T.; Wassie, B.T.; Gold, M.P.; Wang, K.Q.; Miramontes, R.; et al. Aberrant Development Corrected in Adult-Onset Huntington's Disease iPSC-Derived Neuronal Cultures via WNT Signaling Modulation. *Stem Cell Rep.* **2020**, *14*, 406–419, https://doi.org/10.1016/j.stemcr.2020.01.015.
100. Papandreou, A.; McTague, A.; Trump, N.; Ambegaonkar, G.; Ngoh, A.; Meyer, E.; Scott, R.H.; A Kurian, M. *GABRB3*mutations: A new and emerging cause of early infantile epileptic encephalopathy. *Dev. Med. Child Neurol.* **2015**, *58*, 416–420, https://doi.org/10.1111/dmcn.12976.
101. Tanaka, M.; DeLorey, T.M.; Delgado-Escueta, A.V.; Olsen, R.W. GABRB3, Epilepsy, and Neurodevelopment. **2012**, 887–899, https://doi.org/10.1093/med/9780199746545.003.0070.
102. Møller, R.S.; Wuttke, T.V.; Helbig, I.; Marini, C.; Johannesen, K.M.; Brilstra, E.H.; Vaher, U.; Borggraefe, I.; Talvik, I.; Talvik, T.; et al. Mutations in *GABRB3*. *Neurology* **2017**, *88*, 483–492, https://doi.org/10.1212/wnl.0000000000003565.
103. Byrne, L.M.; Rodrigues, F.B.; Blennow, K.; Durr, A.; Leavitt, B.R.; Roos, R.A.C.; I Scahill, R.; Tabrizi, S.J.; Zetterberg, H.; Langbehn, D.; et al. Neurofilament light protein in blood as a potential biomarker of neurodegeneration in Huntington's disease: A retrospective cohort analysis. *Lancet Neurol.* **2017**, *16*, 601–609, https://doi.org/10.1016/s1474-4422(17)30124-2.
104. Johnson, E.B.; Byrne, L.M.; Gregory, S.; Rodrigues, F.B.; Blennow, K.; Durr, A.; Leavitt, B.; Roos, R.A.; Zetterberg, H.; Tabrizi, S.J.; et al. Neurofilament light protein in blood predicts regional atrophy in Huntington disease. *Neurology* **2018**, *90*, e717–e723, https://doi.org/10.1212/wnl.0000000000005005.
105. Rodrigues, F.B., Byrne, L.M., Tortelli, R., Johnson, E.B., Wijeratne, P.A., Arridge, M., de Vita, E., Ghazaleh, N., Houghton, R., Furby, H., Alexander, D.C., Tabrizi, S.J., Schobel, S., Scahill, R.I., Heslegrave, A., Zetterberg, H., & Wild, E.J. (2020). Longitudinal dynamics of mutant huntingtin and neurofilament light in Huntington's disease: The prospective HD-CSF study. In medRxiv. https://doi.org/10.1101/2020.03.31.20045260.
106. Soylu-Kucharz, R.; Sandelius, .; Sjögren, M.; Blennow, K.; Wild, E.J.; Zetterberg, H.; Björkqvist, M. Neurofilament light protein in CSF and blood is associated with neurodegeneration and disease severity in Huntington's disease R6/2 mice. *Sci. Rep.* **2017**, *7*, 1–8, https://doi.org/10.1038/s41598-017-14179-1.
107. Jordanova, A.; De Jonghe, P.; Boerkoel, C.F.; Takashima, H.; De Vriendt, E.; Ceuterick, C.; Martin, J.-J.; Butler, I.J.; Mancias, P.; Ch Papasozomenos, S.; et al. Mutations in the neurofilament light chain gene (NEFL) cause early onset severe Charcot-Marie-Tooth disease. *Brain* **2003**, *126*, 590–597, https://doi.org/10.1093/brain/awg059.
108. Bäckström, D.; Linder, J.; Mo, S.J.; Riklund, K.; Zetterberg, H.; Blennow, K.; Forsgren, L.; Lenfeldt, N. NfL as a biomarker for neurodegeneration and survival in Parkinson disease. *Neurology* **2020**, *95*, e827–e838, https://doi.org/10.1212/wnl.0000000000010084.
109. Chung, D.; Shum, A.; Caraveo, G. GAP-43 and BASP1 in Axon Regeneration: Implications for the Treatment of Neurodegenerative Diseases. *Front. Cell Dev. Biol.* **2020**, *8*, https://doi.org/10.3389/fcell.2020.567537.
110. Boutary, S.; Echaniz-Laguna, A.; Adams, D.; Loisel-Duwattez, J.; Schumacher, M.; Massaad, C.; Massaad-Massade, L. Treating PMP22 gene duplication-related Charcot-Marie-Tooth disease: The past, the present and the future. *Transl. Res.* **2020**, *227*, 100–111, https://doi.org/10.1016/j.trsl.2020.07.006.

111. Langfelder, P.; Cantle, J.P.; Chatzopoulou, D.; Wang, N.; Gao, F.; Al-Ramahi, I.; Lu, X.-H.; Ramos, E.M.; El-Zein, K.; Zhao, Y.; et al. Integrated genomics and proteomics define huntingtin CAG length–dependent networks in mice. *Nat. Neurosci.* **2016**, *19*, 623–633, https://doi.org/10.1038/nn.4256.
112. Peek, S.L.; Mah, K.M.; Weiner, J.A. Regulation of neural circuit formation by protocadherins. *Experientia* **2017**, *74*, 4133–4157, https://doi.org/10.1007/s00018-017-2572-3.
113. Melka, M.G.; A Castellani, C.; Rajakumar, N.; O'Reilly, R.; Singh, S.M. Olanzapine-induced methylation alters cadherin gene families and associated pathways implicated in psychosis. *BMC Neurosci.* **2014**, *15*, 112, https://doi.org/10.1186/1471-2202-15-112.
114. Ament, S.; Pearl, J.R.; Cantle, J.P.; Bragg, R.M.; Skene, P.J.; Coffey, S.R.; E Bergey, D.; Wheeler, V.C.; E MacDonald, M.; Baliga, N.S.; et al. Transcriptional regulatory networks underlying gene expression changes in Huntington's disease. *Mol. Syst. Biol.* **2018**, *14*, e7435, https://doi.org/10.15252/msb.20167435.
115. Kikkawa, T.; Casingal, C.; Chun, S.H.; Shinohara, H.; Hiraoka, K.; Osumi, N. The role of Pax6 in brain development and its impact on pathogenesis of autism spectrum disorder. *Brain Res.* **2018**, *1705*, 95–103, https://doi.org/10.1016/j.brainres.2018.02.041.
116. Mishra, S.; Maurya, S.; Srivastava, K.; Shukla, S.; Mishra, R. Pax6 influences expression patterns of genes involved in neuro-degeneration. *Ann. Neurosci.* **2015**, *22*, 226–231, https://doi.org/10.5214/ans.0972.7531.220407.
117. Chandrasekaran, S.; Bonchev, D. Network analysis of human post-mortem microarrays reveals novel genes, microRNAs, and mechanistic scenarios of potential importance in fighting huntington's disease. *Comput. Struct. Biotechnol. J.* **2016**, *14*, 117–130, https://doi.org/10.1016/j.csbj.2016.02.001.
118. Breen, M.S.; Ozcan, S.; Ramsey, J.M.; Wang, Z.; Ma'Ayan, A.; Rustogi, N.; Gottschalk, M.G.; Webster, M.J.; Weickert, C.S.; Buxbaum, J.D.; et al. Temporal proteomic profiling of postnatal human cortical development. *Transl. Psychiatry* **2018**, *8*, 267, https://doi.org/10.1038/s41398-018-0306-4.
119. Laumonnier, F.; Cuthbert, P.C.; Grant, S.G. The Role of Neuronal Complexes in Human X-Linked Brain Diseases. *Am. J. Hum. Genet.* **2007**, *80*, 205–220, https://doi.org/10.1086/511441.
120. Hui, L.; Slonim, D.; Wick, H.C.; Johnson, K.L.; Koide, K.; Bianchi, D.W. Novel neurodevelopmental information revealed in amniotic fluid supernatant transcripts from fetuses with trisomies 18 and 21. *Qual. Life Res.* **2012**, *131*, 1751–1759, https://doi.org/10.1007/s00439-012-1195-x.
121. Marsh, A.G.; Cottrell, M.T.; Goldman, M.F. Epigenetic DNA Methylation Profiling with MSRE: A Quantitative NGS Approach Using a Parkinson's Disease Test Case. *Front. Genet.* **2016**, *7*, 191, https://doi.org/10.3389/fgene.2016.00191.
122. Kumar, A.; Zhang, J.; Tallaksen-Greene, S.; Crowley, M.R.; Crossman, D.; Morton, A.J.; Van Groen, T.; Kadish, I.; Albin, R.L.; Lesort, M.; et al. Allelic series of Huntington's disease knock-in mice reveals expression discorrelates. *Hum. Mol. Genet.* **2016**, *25*, 1619–1636, https://doi.org/10.1093/hmg/ddw040.
123. Corrêa, T.; Venâncio, A.C.; Galera, M.F.; Riegel, M. Candidate Genes Associated with Delayed Neuropsychomotor Development and Seizures in a Patient with Ring Chromosome 20.. *Case Rep. Genet.* **2020**, *2020*, 5957415–6, https://doi.org/10.1155/2020/5957415.
124. Qureshi, I.A.; Gokhan, S.; Mehler, M.F. REST and CoREST are transcriptional and epigenetic regulators of seminal neural fate decisions. *Cell Cycle* **2010**, *9*, 4477–4486, https://doi.org/10.4161/cc.9.22.13973.
125. Lee, M.T.M.; Chen, C.H.; Lee, C.S.; Chong, M.Y.; Ouyang, W.C.; Chiu, N.Y.; Chuo, L.J.; Tan, H.K.L.; Lane, H.Y.; Chang, T.J.; et al. Genome-wide association study of bipolar I disorder in the Han Chinese population. *Mol. Psychiatry* **2010**, *16*, 548–556, https://doi.org/10.1038/mp.2010.43.
126. Benes, F.M. Amygdalocortical Circuitry in Schizophrenia: From Circuits to Molecules. *Neuropsychopharmacology* **2009**, *35*, 239–257, https://doi.org/10.1038/npp.2009.116.
127. Sibille, E.; Wang, Y.; Joeyen-Waldorf, J.; Gaiteri, C.; Surget, A.; Oh, S.; Belzung, C.; Tseng, G.C.; Lewis, D. A Molecular Signature of Depression in the Amygdala. *Am. J. Psychiatry* **2009**, *166*, 1011–1024, https://doi.org/10.1176/appi.ajp.2009.08121760.
128. Takehashi, M.; Alioto, T.; Stedeford, T.; Persad, A.S.; Banasik, M.; Masliah, E.; Tanaka, S.; Ueda, K. Septin 3 Gene Polymorphism in Alzheimer's Disease. *Gene Expr.* **2003**, *11*, 263–270, https://doi.org/10.3727/000000003783992243.
129. Teuling, E.; Bourgonje, A.; Veenje, S.; Thijssen, K.; De Boer, J.; Van Der Velde, J.; Swertz, M.; Nollen, E. Modifiers of mutant huntingtin aggregationfunctional conservation of C. elegans-modifiers of polyglutamine aggregation. *PLoS Curr.* **2011**, *3*, RRN1255, https://doi.org/10.1371/currents.rrn1255.
130. Carvill, G.L.; Helbig, K.L.; Myers, C.T.; Scala, M.; Huether, R.; Lewis, S.; Kruer, T.N.; Guida, B.S.; Bakhtiari, S.; Sebe, J.; et al. Damaging de novo missense variants inEEF1A2lead to a developmental and degenerative epileptic-dyskinetic encephalopathy. *Hum. Mutat.* **2020**, *41*, 1263–1279, https://doi.org/10.1002/humu.24015.
131. Lance, E.; Kronenbuerger, M.; Cohen, J.S.; Furmanski, O.; Singer, H.S.; Fatemi, A. Successful treatment of choreo-athetotic movements in a patient with an EEF1A2 gene variant. *SAGE Open Med Case Rep.* **2018**, *6*, https://doi.org/10.1177/2050313x18807622.
132. Lin, M.; Pedrosa, E.; Hrabovsky, A.; Chen, J.; Puliafito, B.R.; Gilbert, S.R.; Zheng, D.; Lachman, H.M. Integrative transcriptome network analysis of iPSC-derived neurons from schizophrenia and schizoaffective disorder patients with 22q11.2 deletion. *BMC Syst. Biol.* **2016**, *10*, 1–20, https://doi.org/10.1186/s12918-016-0366-0.
133. Dong, X.; Cong, S. Identification of differentially expressed genes and regulatory relationships in Huntington's disease by bioinformatics analysis. *Mol. Med. Rep.* **2018**, *17*, 4317–4326, https://doi.org/10.3892/mmr.2018.8410.
134. Manap, A.S.A.; Madhavan, P.; Vijayabalan, S.; Chia, A.; Fukui, K. Explicating anti-amyloidogenic role of curcumin and piperine via amyloid beta (Aβ) explicit pathway: Recovery and reversal paradigm effects. *PeerJ* **2020**, *8*, e10003, https://doi.org/10.7717/peerj.10003.

135. Pehlivan, S.; Fedakar, R.; Eren, B.; Akyol, S.; Eren, F.; Inanir, N.T.; Gurses, M.S.; Ural, M.N.; Tagil, S.M.; Demircan, K. ADAMTS4, 5, 9, and 15 Expressions in the Autopsied Brain of Patients with Alzheimer's Disease: A Preliminary Immunohistochemistry Study. **2016**, *26*, 7–14, https://doi.org/10.5455/bcp.20150706034008.
136. Hong, C.C.; Tang, A.T.; Detter, M.R.; Choi, J.P.; Wang, R.; Yang, X.; Guerrero, A.A.; Wittig, C.F.; Hobson, N.; Girard, R.; et al. Cerebral cavernous malformations are driven by ADAMTS5 proteolysis of versican. *J. Exp. Med.* **2020**, *217*, https://doi.org/10.1084/jem.20200140.
137. Zhou, J.; Zhang, W.; Wei, C.; Zhang, Z.; Yi, D.; Peng, X.; Peng, J.; Yin, R.; Zheng, Z.; Qi, H.; et al. Weighted correlation network bioinformatics uncovers a key molecular biosignature driving the left-sided heart failure. *BMC Med Genom.* **2020**, *13*, 1–13, https://doi.org/10.1186/s12920-020-00750-9.
138. Waters, C.W.; Varuzhanyan, G.; Talmadge, R.J.; Voss, A.A. Huntington disease skeletal muscle is hyperexcitable owing to chloride and potassium channel dysfunction. *Proc. Natl. Acad. Sci.* **2013**, *110*, 9160–9165, https://doi.org/10.1073/pnas.1220068110.
139. Chandrasekaran, S.; Bonchev, D. A NETWORK VIEW ON PARKINSON'S DISEASE. *Comput. Struct. Biotechnol. J.* **2013**, *7*, e201304004, https://doi.org/10.5936/csbj.201304004.
140. Chiang, M.-C.; Chen, H.-M.; Lee, Y.-H.; Chang, H.-H.; Wu, Y.-C.; Soong, B.-W.; Chen, C.-M.; Wu, Y.-R.; Liu, C.-S.; Niu, D.-M.; et al. Dysregulation of C/EBPα by mutant Huntingtin causes the urea cycle deficiency in Huntington's disease. *Hum. Mol. Genet.* **2007**, *16*, 483–498, https://doi.org/10.1093/hmg/ddl481.
141. Jeong, H.; Moye, L.S.; Southey, B.R.; Hernandez, A.G.; Dripps, I.; Romanova, E.V.; Rubakhin, S.S.; Sweedler, J.V.; Pradhan, A.A.; Rodriguez-Zas, S.L. Gene Network Dysregulation in the Trigeminal Ganglia and Nucleus Accumbens of a Model of Chronic Migraine-Associated Hyperalgesia. *Front. Syst. Neurosci.* **2018**, *12*, https://doi.org/10.3389/fnsys.2018.00063.
142. Miller, J.R.C.; Lo, K.K.; Andre, R.; Moss, D.J.H.; Träger, U.; Stone, T.C.; Jones, L.; Holmans, P.; Plagnol, V.; Tabrizi, S.J. RNA-Seq of Huntington's disease patient myeloid cells reveals innate transcriptional dysregulation associated with proinflammatory pathway activation. *Hum. Mol. Genet.* **2016**, *25*, 2893–2904, https://doi.org/10.1093/hmg/ddw142.
143. Li, J.; Khankan, R.R.; Caneda, C.; Godoy, M.I.; Haney, M.S.; Krawczyk, M.C.; Bassik, M.C.; Sloan, S.A.; Zhang, Y. Astrocyte-to-astrocyte contact and a positive feedback loop of growth factor signaling regulate astrocyte maturation.. *Glia* **2019**, *67*, 1571–1597, https://doi.org/10.1002/glia.23630.
144. Agostoni, E.; Michelazzi, S.; Maurutto, M.; Carnemolla, A.; Ciani, Y.; Vatta, P.; Roncaglia, P.; Zucchelli, S.; Leanza, G.; Mantovani, F.; et al. Effects of Pin1 Loss in HdhQ111 Knock-in Mice. *Front. Cell. Neurosci.* **2016**, *10*, 110–110, https://doi.org/10.3389/fncel.2016.00110.
145. Wang, F.; Yang, Y.; Lin, X.; Wang, J.-Q.; Wu, Y.-S.; Xie, W.; Wang, D.; Zhu, S.; Liao, Y.-Q.; Sun, Q.; et al. Genome-wide loss of 5-hmC is a novel epigenetic feature of Huntington's disease. *Hum. Mol. Genet.* **2013**, *22*, 3641–3653, https://doi.org/10.1093/hmg/ddt214.
146. Salvalaio, M.; D'Avanzo, F.; Rigon, L.; Zanetti, A.; D'Angelo, M.; Valle, G.; Scarpa, M.; Tomanin, R. Brain RNA-Seq Profiling of the Mucopolysaccharidosis Type II Mouse Model. *Int. J. Mol. Sci.* **2017**, *18*, 1072, https://doi.org/10.3390/ijms18051072.
147. Yamanaka, T.; Wong, H.K.; Tosaki, A.; Bauer, P.O.; Wada, K.; Kurosawa, M.; Shimogori, T.; Hattori, N.; Nukina, N. Large-Scale RNA Interference Screening in Mammalian Cells Identifies Novel Regulators of Mutant Huntingtin Aggregation. *PLoS ONE* **2014**, *9*, e93891, https://doi.org/10.1371/journal.pone.0093891.
148. Strand, A.D.; Aragaki, A.K.; Shaw, D.; Bird, T.; Holton, J.; Turner, C.; Tapscott, S.J.; Tabrizi, S.J.; Schapira, A.H.; Kooperberg, C.; et al. Gene expression in Huntington's disease skeletal muscle: A potential biomarker. *Hum. Mol. Genet.* **2005**, *14*, 1863–1876, https://doi.org/10.1093/hmg/ddi192.
149. Li, M.D.; Burns, T.C.; A Morgan, A.; Khatri, P. Integrated multi-cohort transcriptional meta-analysis of neurodegenerative diseases. *Acta Neuropathol. Commun.* **2014**, *2*, 1–23, https://doi.org/10.1186/s40478-014-0093-y.
150. Moradifard, S.; Hoseinbeyki, M.; Ganji, S.M.; Minuchehr, Z. Analysis of microRNA and Gene Expression Profiles in Alzheimer's Disease: A Meta-Analysis Approach. *Sci. Rep.* **2018**, *8*, 1–17, https://doi.org/10.1038/s41598-018-20959-0.
151. The HD iPSC Consortium Induced Pluripotent Stem Cells from Patients with Huntington's Disease Show CAG-Repeat-Expansion-Associated Phenotypes. *Cell Stem Cell* **2012**, *11*, 264–278, https://doi.org/10.1016/j.stem.2012.04.027.
152. Loera-Valencia, R.; Piras, A.; Ismail, M.A.M.; Manchanda, S.; Eyjolfsdottir, H.; Saido, T.C.; Johansson, J.; Eriksdotter, M.; Winblad, B.; Nilsson, P. Targeting Alzheimer's disease with gene and cell therapies. *J. Intern. Med.* **2018**, *284*, 2–36, https://doi.org/10.1111/joim.12759.
153. Tao, B.; Ling, Y.; Zhang, Y.; Li, S.; Zhou, P.; Wang, X.; Li, B.; Jun, Z.; Zhang, W.; Xu, C.; et al. CA 10 and CA 11 negatively regulate neuronal activity-dependent growth of gliomas. *Mol. Oncol.* **2019**, *13*, 1018–1032, https://doi.org/10.1002/1878-0261.12445.
154. Krüger, R.; Schöls, L.; Müller, T.; Kuhn, W.; Woitalla, D.; Przuntek, H.; Epplen, J.T.; Riess, O. Evaluation of the γ-synuclein gene in German Parkinson's disease patients. *Neurosci. Lett.* **2001**, *310*, 191–193, https://doi.org/10.1016/s0304-3940(01)02127-9.
155. Mahinrad, S.; Bulk, M.; van der Velpen, I.; Mahfouz, A.; van Roon-Mom, W.; Fedarko, N.; Yasar, S.; Sabayan, B.; van Heemst, D.; van der Weerd, L. Natriuretic Peptides in Post-mortem Brain Tissue and Cerebrospinal Fluid of Non-demented Humans and Alzheimer's Disease Patients. *Front. Neurosci.* **2018**, *12*, 864, https://doi.org/10.3389/fnins.2018.00864.
156. Scholl-Bürgi, S.; Sigl, S.B.; Häberle, J.; Haberlandt, E.; Rostásy, K.; Ertl, C.; Eichinger-Öttl, U.; Heinz-Erian, P.; Karall, D. Amino acids in CSF and plasma in hyperammonaemic coma due to arginase1 deficiency. *J. Inherit. Metab. Dis.* **2008**, *31*, 323–328, https://doi.org/10.1007/s10545-008-0903-0.
157. Fridén, M.; Winiwarter, S.; Jerndal, G.; Bengtsson, O.; Wan, H.; Bredberg, U.; Hammarlund-Udenaes, M.; Antonsson, M. Structure−Brain Exposure Relationships in Rat and Human Using a Novel Data Set of Unbound Drug Concentrations in Brain Interstitial and Cerebrospinal Fluids. *J. Med. Chem.* **2009**, *52*, 6233–6243, https://doi.org/10.1021/jm901036q.

158. Pellegrini, L.; Bonfio, C.; Chadwick, J.; Begum, F.; Skehel, M.; Lancaster, M.A. Human CNS barrier-forming organoids with cerebrospinal fluid production. *Science* **2020**, *369*, https://doi.org/10.1126/science.aaz5626.
159. Elmenhorst, D.; Meyer, P.T.; Matusch, A.; Winz, O.H.; Bauer, A. Caffeine Occupancy of Human Cerebral A_1 Adenosine Receptors: In Vivo Quantification with ^{18}F-CPFPX and PET. *J. Nucl. Med.* **2012**, *53*, 1723–1729, https://doi.org/10.2967/jnumed.112.105114.
160. Johannessen, S.; Gerna, M.; Bakke, J.; Strandjord, R.; Morselli, P. CSF CONCENTRATIONS AND SERUM PROTEIN BINDING OF CARBAMAZEPINE AND CARBAMAZEPINE-10, 11-EPOXIDE IN EPILEPTIC PATIENTS. *Br. J. Clin. Pharmacol.* **1976**, *3*, 575–582, https://doi.org/10.1111/j.1365-2125.1976.tb04878.x.
161. Paulzen, M.; Lammertz, S.E.; Gründer, G.; Veselinovic, T.; Hiemke, C.; Tauber, S.C. Measuring citalopram in blood and central nervous system. *Int. Clin. Psychopharmacol.* **2016**, *31*, 119–126, https://doi.org/10.1097/yic.0000000000000114.
162. Allonen, H.; Andersson, K.-E.; Iisalo, E.; Kanto, J.; Strömblad, L.; Wettrell, G. Passage of Digoxin into Cerebrospinal Fluid in Man. *Acta Pharmacol. et Toxicol.* **2009**, *41*, 193–202, https://doi.org/10.1111/j.1600-0773.1977.tb02139.x.
163. Pardridge, W.M. CSF, blood-brain barrier, and brain drug delivery. *Expert Opin. Drug Deliv.* **2016**, *13*, 963–975, https://doi.org/10.1517/17425247.2016.1171315.
164. González-Sánchez, M.; Jiménez, J.; Narváez, A.; Antequera, D.; Llamas-Velasco, S.; Martín, A.H.-S.; Arjona, J.A.M.; De Munain, A.L.; Bisa, A.L.; Marco, M.-P.; et al. Kynurenic Acid Levels are Increased in the CSF of Alzheimer’s Disease Patients. *Biomolecules* **2020**, *10*, 571, doi:10.3390/biom10040571.
165. Vassal, G.; Valteau, D.; Bonnay, M.; Patte, C.; Aubier, F.; Lemerle, J. Cerebrospinal Fluid and Plasma Methotrexate Levels Following High-Dose Regimen Given as a 3-Hour Intravenous Infusion in Children with Nonhodgkin's Lymphoma. *Pediatr. Hematol. Oncol.* **1990**, *7*, 71–77, https://doi.org/10.3109/08880019009034320.
166. Ogutu, B.R.; Newton, C.; Muchohi, S.N.; Otieno, G.O.; Edwards, G.; Watkins, W.M.; Kokwaro, G.O. Pharmacokinetics and clinical effects of phenytoin and fosphenytoin in children with severe malaria and status epilepticus. *Br. J. Clin. Pharmacol.* **2003**, *56*, 112–119, https://doi.org/10.1046/j.1365-2125.2003.01829.x.
